# Motion-compensated scheme for sequential scanned statistical iterative dual-energy CT reconstruction

**DOI:** 10.1088/1361-6560/acdf38

**Published:** 2023-07-05

**Authors:** Tao Ge, Rui Liao, Maria Medrano, David G Politte, Bruce R Whiting, Jeffrey F Williamson, Joseph A O’Sullivan

**Affiliations:** 1 Washington University in St. Louis, Saint Louis, MO, 63130, United States of America; 2 University of Pittsburgh, Pittsburgh, PA, 15260, United States of America

**Keywords:** dual-energy CT, image registration, CT reconstruction, material decomposition, iterative reconstruction

## Abstract

*Objective.* Dual-energy computed tomography (DECT) has been widely used to reconstruct numerous types of images due its ability to better discriminate tissue properties. Sequential scanning is a popular dual-energy data acquisition method as it requires no specialized hardware. However, patient motion between two sequential scans may lead to severe motion artifacts in DECT statistical iterative reconstructions (SIR) images. The objective is to reduce the motion artifacts in such reconstructions. *Approach.* We propose a motion-compensation scheme that incorporates a deformation vector field into any DECT SIR. The deformation vector field is estimated via the multi-modality symmetric deformable registration method. The precalculated registration mapping and its inverse or adjoint are then embedded into each iteration of the iterative DECT algorithm. *Main results.* Results from a simulated and clinical case show that the proposed framework is capable of reducing motion artifacts in DECT SIRs. Percentage mean square errors in regions of interest in the simulated and clinical cases were reduced from 4.6% to 0.5% and 6.8% to 0.8%, respectively. A perturbation analysis was then performed to determine errors in approximating the continuous deformation by using the deformation field and interpolation. Our findings show that errors in our method are mostly propagated through the target image and amplified by the inverse matrix of the combination of the Fisher information and Hessian of the penalty term. *Significance.* We have proposed a novel motion-compensation scheme to incorporate a 3D registration method into the joint statistical iterative DECT algorithm in order to reduce motion artifacts caused by inter-scan motion, and successfully demonstrate that interscan motion corrections can be integrated into the DECT SIR process, enabling accurate imaging of radiological quantities on conventional SECT scanners, without significant loss of either computational efficiency or accuracy.

## 1. Introduction

Compared to conventional single-energy CT (SECT), dual-energy CT (DECT) generates more informative and quantitative results from transmission sinograms acquired at two different x-ray spectra. In 1976, Alvarez and Macovski (1976) represented the linear attenuation coefficient (LAC) as the outer product of an energy-dependent basis function and a set of spatially-dependent coefficients and introduced the basic idea of multi-energy CT to estimate the energy-independent spatial functions.

Since then, DECT has been further developed and is widely used in numerous clinical applications, including automated bone removal in CT angiography, blood-pool imaging, and virtual noncontrastenhanced imaging (McCollough *et al* 2015). In radiotherapy applications, DECT has improved the accuracy with which electron density (Tsunoo *et al* 2008), effective atomic number (Goodsitt *et al* 2011, Bonnin *et al* 2014, Hua *et al* 2018, Schaeffer *et al* 2021) and proton stopping power (Han *et al* 2016, Taasti *et al* 2016) can be imaged. More recently, statistical iterative reconstruction (SIR) algorithms for DECT have been introduced to reduce noise and improve resolution and accuracy (Fessler *et al* 2002, O’Sullivan *et al* 2004, Zhang *et al* 2014, Chen *et al* 2016, Zhang *et al* 2017). A novel SIR algorithm that jointly operates on different-energy raw-data sinograms demonstrated that it achieves sub-percentage uncertainty in imaging proton stopping-power (Zhang *et al* 2019, Medrano *et al* 2020, 2022). In current proton-therapy clinical practice, SECT is used to map stopping-power ratios, leading to 2–3.5% proton beam range uncertainty (Paganetti 2012, Park *et al* 2012).

Dual-energy data acquisition methods include rapid-kVp switching scanning, single-source multi-layer scanning, and dual-source scanning (McCollough *et al* 2015), all of which require specialized hardware. In contrast, single-source sequential scanning can be implemented on any conventional SECT scanner. However, this method is more vulnerable to motion and deformation of the scan subject during the scanning process. Such patient motion includes changes in position, respiratory- and cardiac-induced organ motion, peristalsis, and intestinal gas transit, and can be as large as several cm over a 1–10 min period and are highly nonlinear and localized (Langen and Jones 2001). Since the two sinograms are acquired from differently deformed or positioned instances of the same anatomy, potentially large global artifacts result, significantly degrading the quantitative accuracy achieved by DECT SIR.

To address the dual-energy motion artifact, image registration has been incorporated with medical imaging techniques to reconstruct dual-energy images from measurements taken in different patient positions. Gang *et al*, Huang *et al* and Leng have all deployed deformable image registration (DIR) methods to align two sequentially scanned images at different energies before dual-energy material decomposition (Gang *et al* 2009, Leng *et al* 2015, Huang *et al* 2020) and demonstrated significant reduction of motion artifacts. However, these motion-compensated methods are designed for image-domain decomposition techniques, which cannot be simply applied to DECT SIR.

More relevant to our setting, is the literature on single-energy, motion-compensated 4D cone-beam CT (CBCT) image reconstruction. In this application, the source and flat-panel detector assembly slowly rotate (15–60 s/rotation) around the free-breathing patient, acquiring as many as 2000 projections. To minimize image blur and undersampling artifacts, Rit *et al*, followed by many others (Tang *et al* 2012, Brehm *et al* 2013, Mory *et al* 2016), proposed incorporating a 4D inverse deformation vector field (DVF) model into the backprojection operator so that all projections, regardless of breathing phase, can be backprojected onto a single artifact-free reference phase image. Several investigators (Jailin *et al* 2021, Wang and Gu 2013, Renders *et al* 2021) developed iterative 4D CBCT reconstruction algorithms, in which precomputed or iteratively updated 4D deformation fields are incorporated into each iterative image update.

Inspired by these investigations, we propose to incorporate a 3D registration method into our joint statistical iterative DECT algorithm in order to reduce motion artifacts caused by inter-scan motion. To the best of our knowledge, embedding DIR in iterative DECT algorithms has not been previously reported. The proposed scheme is derived from the forward model for a deformed object and can be used by any DECT SIR. As well as testing the method on digital phantoms and patient images, we perform a first-order perturbation analysis to theoretically evaluate the estimation error introduced by registration target-registration errors (TREs).

We acknowledge that intrascan motion can also degrade DECT performance. However, in this work, we consider only the motion between two scans. Nevertheless, 4D-CT motion-compensated DVFs could be incorporated into the proposed scheme. Similarly, for more than two scans, the techniques here can be extended in a straightforward manner to more than two energies.

## 2. Materials and methods

### 2.1. General framework

**Figure 1.**
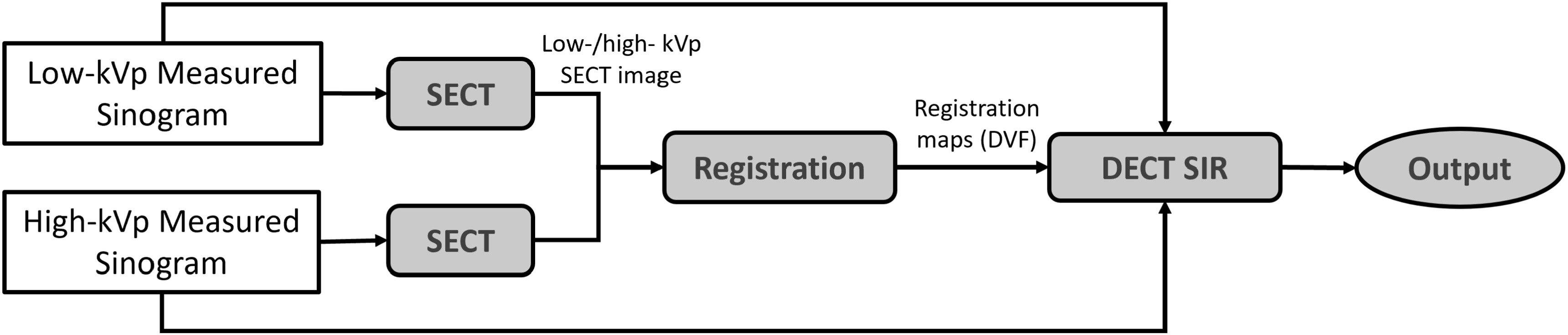
Flowchart of the proposed framework.

As shown in figure 1, the framework of the proposed DECT reconstruction algorithm with registration consists of 2 steps: registration maps evaluation and DECT algorithm.

**Figure 2.**
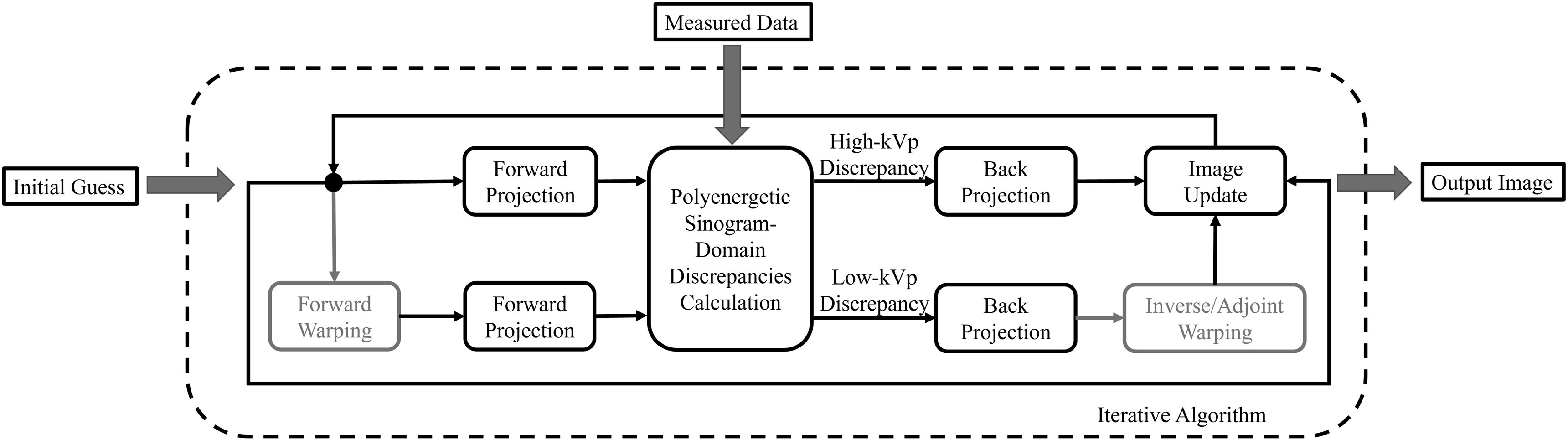
Flowchart of iterative DECT algorithm with registration.

The registration mappings are evaluated based on SECT images reconstructed from low- and high-energy data. The SECT images could be reconstructed by analytical algorithms or iterative algorithms. Single-energy alternating minimization algorithm is utilized in this work to reduce the influence of artifacts, so the registration algorithm is less likely to attempt to match the noise.

After the image registration, the iterative DECT algorithm is initialized by the analytical DECT images, and the deformation fields correct the system operator that maps the data domain and the image domain in both the initialization method and the iterative DECT algorithm.

### 2.2. Algorithm derivation from the forward model

Let ${{\boldsymbol{x}}}_{H}\in {{\mathbb{R}}}^{3}$ and ${{\boldsymbol{x}}}_{L}\in {{\mathbb{R}}}^{3}$ be the coordinate systems describing the subject anatomy during high- and low-energy scanning, respectively. The true deformation transform ${{\boldsymbol{\varphi }}}_{0}:{{\mathbb{R}}}^{3}\to {{\mathbb{R}}}^{3}$, identifies the location ***x***_*L*_ = ***φ***_0_(***x***_*H*_) in the low energy image corresponding to the high-energy image location ***x***_*H*_. ***φ***_0_(***x***_*H*_) is closely related to the DVF ***u***_*L*→*H*_(***x***_*H*_) by ***φ***_0_(***x***_*H*_) = ***u***_*L*→*H*_(***x***_*H*_) + ***x***_*H*_. Given the LAC map ***μ*** (***x***_*H*_, ***E***) as a function of energy *E*, specified in the fixed coordinate system ***x***_*H*_, the warped moving image can be written as ***μ*** (***φ***_0_(***x***_*H*_), ***E***) = (***μ*** ◦ ***φ***_0_)(***x***_*H*_, ***E***), where ◦denotes the function composition operator.

Therefore, the transmission measurements for DECT are modeled by

\begin{eqnarray*}{{\boldsymbol{d}}}_{L}({\boldsymbol{y}})\sim {\int }_{{N}_{E}}{I}_{0,L}({\boldsymbol{y}},{\boldsymbol{E}}){e}^{-\int {\boldsymbol{h}}({{\boldsymbol{x}}}_{H},{\boldsymbol{y}})({\boldsymbol{\mu }}\circ {{\boldsymbol{\varphi }}}_{0})({{\boldsymbol{x}}}_{H},{\boldsymbol{E}})d{{\boldsymbol{x}}}_{H}}d{\boldsymbol{E}},\end{eqnarray*}

\begin{eqnarray*}{{\boldsymbol{d}}}_{H}({\boldsymbol{y}})\sim {\int }_{{N}_{E}}{I}_{0,H}({\boldsymbol{y}},{\boldsymbol{E}}){e}^{-\int {\boldsymbol{h}}({{\boldsymbol{x}}}_{H},{\boldsymbol{y}}){\boldsymbol{\mu }}({{\boldsymbol{x}}}_{H},{\boldsymbol{E}})d{{\boldsymbol{x}}}_{H}}d{\boldsymbol{E}},\end{eqnarray*}

where *d*_*L*_ and *d*_*H*_ denote the low- and high- energy measurements, respectively, ${\boldsymbol{y}}\in {{\mathbb{R}}}^{3}$ denotes the location in the measurement space, *N*_*E*_ is the domain of the energy bin, *I*_0_ denotes the photon counting number in the absence of the object (explicitly the outer product of the bowtie filter in the sinogram domain and the spectrum in the energy domain), and ***h*** (***x***, ***y***) denotes the system operator.

Since ***x***_*L*_ = ***φ***_0_(***x***_*H*_) and ${{\boldsymbol{x}}}_{H}={{\boldsymbol{\varphi }}}_{0}^{-1}({{\boldsymbol{x}}}_{L})$, the forward-projection of an image ***μ*** (***x***_*H*_, ***E***) from the distorted image frame of reference can be rewritten as

\begin{eqnarray*}{\boldsymbol{FP}}({\boldsymbol{y}}| {\boldsymbol{\mu }}({{\boldsymbol{x}}}_{L},{\boldsymbol{E}}))=\int {\boldsymbol{\mu }}({{\boldsymbol{x}}}_{L},{\boldsymbol{E}})\int {\boldsymbol{h}}({{\boldsymbol{x}}}_{H},{\boldsymbol{y}})\delta ({{\boldsymbol{\varphi }}}_{0}({{\boldsymbol{x}}}_{H})-{{\boldsymbol{x}}}_{L})d{{\boldsymbol{x}}}_{H}d{{\boldsymbol{x}}}_{L},\end{eqnarray*}

where the Dirac-delta function *δ* satisfies ${\int }_{{{ \mathcal B }}_{\epsilon }(0)}\delta ({\boldsymbol{x}})d{\boldsymbol{x}}\,=\,1$ for all open balls ${{ \mathcal B }}_{\epsilon }(0)\in {{\mathbb{R}}}^{3}$ of radius *ϵ* > 0, centering the origin.

The function $h^{\prime} ({{\boldsymbol{x}}}_{L},{\boldsymbol{y}})=\int {\boldsymbol{h}}({{\boldsymbol{x}}}_{H},{\boldsymbol{y}})\delta ({{\boldsymbol{\varphi }}}_{0}({{\boldsymbol{x}}}_{H})-{{\boldsymbol{x}}}_{L})d{{\boldsymbol{x}}}_{H}$ is the distorted anatomy system operator, which allows an image formed in the high-energy coordinate system to be compared with its low energy projection, which was acquired with the patient in the deformed state. By the definition of adjoint, we can derive the corresponding back-projection as

\begin{eqnarray*}\int h^{\prime} ({{\boldsymbol{x}}}_{L},{\boldsymbol{y}}){\boldsymbol{g}}({\boldsymbol{y}})d{\boldsymbol{y}}\end{eqnarray*}

\begin{eqnarray*}=\int \int {\boldsymbol{h}}({{\boldsymbol{x}}}_{H},{\boldsymbol{y}})\delta ({{\boldsymbol{\varphi }}}_{0}({{\boldsymbol{x}}}_{H})-{{\boldsymbol{x}}}_{L})d{{\boldsymbol{x}}}_{H}{\boldsymbol{g}}({\boldsymbol{y}})d{\boldsymbol{y}}\end{eqnarray*}

\begin{eqnarray*}=\left({\boldsymbol{BP}}\circ {{\boldsymbol{\varphi }}}_{0}^{-1}\right)({{\boldsymbol{x}}}_{L})\left|\displaystyle \frac{d{{\boldsymbol{\varphi }}}_{0}^{-1}({{\boldsymbol{x}}}_{L})}{d{{\boldsymbol{x}}}_{L}}\right|,\end{eqnarray*}

where ***BP*** (***x***_*H*_) = ∫ ***h*** (***x***_*H*_, ***y***) ***g*** (***y***)*d* ***y*** is the back-projection of the sinogram ***g*** (***y***) into the high-energy image domain.

The determinant of the Jacobian of the deformation field ${{\boldsymbol{\varphi }}}_{0}^{-1}$ compensates for the variation of the space density in the epsilon-ball about the location ***x*** after the deformation field is applied. If the neighborhood of the point ***x*** is concentrated when the image is warped by the forward deformation field ***φ***, the point ***x*** tends to get more weight in the gradient calculation.

Because images and sinogram data are discretized, *x*, *y* can be treated as integer indices, i.e. so that image *μ*(*x*, *E*) can be represented as a matrix since we choose to discretize energy as well. In this case, *μ*(*φ*_0_(*x*_*H*_), *E*) is not defined for ${\varphi }_{0}({x}_{H})\notin {\mathbb{Z}}$.

The forward model can be represented as

\begin{eqnarray*}{d}_{L}(y)\sim \displaystyle \sum _{E\in {N}_{E}}{I}_{0,L}(y,E){e}^{-\displaystyle \sum _{{x}_{H}}h({x}_{H},y)\mathrm{Interp}({\varphi }_{0}({x}_{H}):\mu (\cdot ,E))},\end{eqnarray*}

and

\begin{eqnarray*}{d}_{H}(y)\sim \displaystyle \sum _{E\in {N}_{E}}{I}_{0,H}(y,E){e}^{-\displaystyle \sum _{{x}_{H}}h({x}_{H},y)\mu ({x}_{H},E)},\end{eqnarray*}

where $x,y\in {{\mathbb{Z}}}^{3}$ denote the discrete indices of the image space and measurement space, respectively, *μ* denotes the discretized image that only takes integer indices, *μ*( · , *E*) is the attenuation image at the energy *E*, and Interp(***x***: *I*) denotes the interpolated image that is indexed by ${\boldsymbol{x}}\in {{\mathbb{R}}}^{3}$, estimated from the digital image *I*.

The first proposed method for the discretized problem could be easily derived following the continuous case. The back-projection becomes

\begin{eqnarray*}\left({\boldsymbol{BP}}\circ {{\boldsymbol{\varphi }}}_{0}^{-1}\right)({{\boldsymbol{x}}}_{L})\left|\displaystyle \frac{d{{\boldsymbol{\varphi }}}_{0}^{-1}({{\boldsymbol{x}}}_{L})}{d{{\boldsymbol{x}}}_{L}}\right|\approx \mathrm{Interp}({\varphi }_{0}^{-1}({x}_{L}):{BP})\left|\displaystyle \frac{d{{\boldsymbol{\varphi }}}_{0}^{-1}({{\boldsymbol{x}}}_{L})}{d{{\boldsymbol{x}}}_{L}}\right|,\end{eqnarray*}

Note that (9) provides the adjoint operator for an arbitrary interpolation method, which allows the use of nonlinear interpolations in the proposed scheme.

Conventionally, the interpolated image could be computed elementwise by a linear combination of the neighboring value and a set of coefficients

\begin{eqnarray*}\mathrm{Interp}({{\boldsymbol{x}}}_{H}:I)=\displaystyle \sum _{{x}_{L}\in { \mathcal N }({{\boldsymbol{x}}}_{H})}w({{\boldsymbol{x}}}_{H},{x}_{L})I({x}_{L}).\end{eqnarray*}

where ${ \mathcal N }({{\boldsymbol{x}}}_{H})$ denotes the neighborhood indices of *x*_*H*_, and *w*(***x***_*H*_, *x*_*L*_) denotes the coefficients based on the distance between ***x***_*H*_ and *x*_*L*_.

Moreover, for any interpolation of the form in (10), one could combine the deformation field and the interpolation as

\begin{eqnarray*}\mathrm{Interp}({\varphi }_{0}({x}_{H}):\mu (\cdot ,E))=\displaystyle \sum _{{x}_{L}}{w}_{\varphi }(x,{x}_{L})\mu ({x}_{L},E).\end{eqnarray*}

Then, the adjoint of the combination of interpolation and forward-projection to calculate the gradient could also be computed as

\begin{eqnarray*}\displaystyle \sum _{y}\left(\displaystyle \sum _{x}h({x}_{H},y){w}_{\varphi }({x}_{H},{x}_{L})\right)g(y)=\displaystyle \sum _{{x}_{H}}\left(\displaystyle \sum _{y}h({x}_{H},y)g(y)\right){w}_{\varphi }({x}_{H},{x}_{L}),\end{eqnarray*}

which is the back-projection followed by the adjoint of the warped linear interpolation.

### 2.3. DECT SIR with image registration

In the previous section, we have shown that the warp of the image could be plugged into the forward-projection process to form a ‘distorted forward-projection’, and the adjoint of the distorted forward-projection could be represented as a back-projection followed by the inverse warping multiplied by the determinant of its Jacobian matrix (shown in (9)) or the adjoint of the interpolation if the interpolation function is linear (shown in (12)).

Since the substitution of the forward-projection does not influence the convergence property of the original algorithm, one could easily apply the proposed framework to any DECT SIR by warping the image before the target forward-projections and applying the adjoint warping after the corresponding back-projections.

This study employed a material decomposition model to simulate the target LAC, based on the assumption that the LAC of typical biological media can be accurately represented through a linear combination of distinct materials, denoted as $\mu (x,E)={\sum }_{i\,=\,1}^{2}{\mu }_{i}(E){c}_{i}(x)$, where *c* is the basis component weight, and *i* denotes the index of the basis. For the sake of simplicity, here we transform *c*_1_ and *c*_2_ into a 1-dimensional vector, denoting as $C\in {{\mathbb{R}}}^{2{N}_{X}}$. Then, suppose the objective function of DECT SIR has the form of (O’Sullivan and Benac 2007, Huh and Fessler 2009, Xu *et al* 2009, Long and Fessler 2014, Zhang *et al* 2014, Chen *et al* 2016)

\begin{eqnarray*}{\ell }(C)=\displaystyle \sum _{j}\displaystyle \sum _{y}{ \mathcal D }\left({d}_{j}(y),{g}_{j}(y:C)\right)+\lambda { \mathcal R }(C),\end{eqnarray*}

where ${ \mathcal D }:({{\mathbb{R}}}^{2{N}_{y}},{{\mathbb{R}}}^{2{N}_{y}})\to {\mathbb{R}}$ denotes the data fidelity term, ${ \mathcal R }:{{\mathbb{R}}}^{2{N}_{X}}\to {\mathbb{R}}$ denotes the regularization term, and *λ* is a scalar that controls the penalty strength; *j* ∈ [*L*, *H*] denotes the scan index, and ${\left[{g}_{j}(C)\right]}_{y}$ is the mean photon counts estimated from the basis images *C*. The update step can be written as a function involving backprojections of the reweighted mean sinograms and the reweighted measured sinograms,

\begin{eqnarray*}{C}_{{\mathrm{new}}}+=f\left(C,{H}^{\dagger }{S}_{L}(C)+{H}^{\dagger }{S}_{H}(C),{H}^{\dagger }{P}_{L}({d}_{L},C)+{H}^{\dagger }{P}_{H}({d}_{H},C),\lambda { \mathcal R }(C)\right),\end{eqnarray*}

where

\begin{eqnarray*}{q}_{j}(y,E:C)={I}_{0,j}\exp \left(-\displaystyle \sum _{i}{\mu }_{i}(E){\left[{HC}\right]}_{i,y}\right),\end{eqnarray*}

\begin{eqnarray*}{g}_{j}(y,E:C)=\displaystyle \sum _{E}{q}_{j}(y,E:C),\end{eqnarray*}

\begin{eqnarray*}{\left[{S}_{j}(C)\right]}_{(i,y)}=\displaystyle \sum _{E}{\mu }_{i}(E){q}_{j}(y,E:C),\end{eqnarray*}

\begin{eqnarray*}{\left[{P}_{j}({d}_{j},C)\right]}_{(i,y)}={d}_{j}(y)\displaystyle \frac{{\sum }_{E}{q}_{j}(y,E:C){\mu }_{i}(E)}{{\sum }_{E^{\prime} }{q}_{j}(y,E:C)},\end{eqnarray*}

$H\in {{\mathbb{R}}}^{2{N}_{Y}\times 2{N}_{X}}$ and ${H}^{\dagger }\in {{\mathbb{R}}}^{2{N}_{X}\times 2{N}_{Y}}$ denote the system operator (forward-projection) and its adjoint (back-projection), respectively, *N*_*X*_ and *N*_*Y*_ are the numbers of measurements and image voxels, respectively. Two ${{\mathbb{R}}}^{{N}_{Y}\times {N}_{X}}$ matrices on the diagonal of *H* are identical, and the ${{\mathbb{R}}}^{{N}_{Y}\times {N}_{X}}$ matrices off the diagonal of *H* are filled with zeros. Therefore, *HC*, *S*_*j*_(*C*) and *P*_*j*_(*d*_*j*_, *C*) are ${{\mathbb{R}}}^{2{N}_{Y}\times 1}$ vectors. $\exp (\cdot )$ denotes the element-wise exponentiation, $f:{{\mathbb{R}}}^{{N}_{Y}}\to {{\mathbb{R}}}^{{N}_{Y}}$ is the gradient function that evaluates the update direction based on the back-projections of the mean and measured transmissions as well as the penalty term.

The proposed method changes the updating step from (14) to

\begin{eqnarray*}\begin{array}{c}{C}_{{\mathrm{new}}}+=f\left(C,{\tilde{W}}_{\varphi }({H}^{\dagger }{S}_{L}({W}_{\varphi }(C))),{H}^{\dagger }{S}_{H}(C),\right.\\ \,\left.\times {\tilde{W}}_{\varphi }({H}^{\dagger }{P}_{L}({d}_{L},{W}_{\varphi }(C))),{H}^{\dagger }{P}_{H}({d}_{H},C),\lambda { \mathcal R }(C)\right),\end{array}\end{eqnarray*}

where ${W}_{\varphi }\in {{\mathbb{R}}}^{2{N}_{X}}\to {{\mathbb{R}}}^{2{N}_{X}}$ is the interpolation operator given the deformation field *φ*, $\tilde{{W}_{\varphi }}$ is a dummy operator that represents ${W}_{{\varphi }^{-1}}$ or ${W}_{\varphi }^{\dagger }$, and ${W}_{\varphi }^{\dagger }$ is the adjoint operator of *W*_*φ*_ if *W*_*φ*_ represents a linear operation. The flowchart is shown in figure 2.

### 2.4. Error analysis

Due to the inherent errors in approximating a deformation using the combination of the warping and the interpolation, there will always be errors between the ground truth and the computed image. In this section, we theoretically analyze the influence of warping error on the result of DECT SIR.

Let *C* ^*f*^ be the stationary point of the warped DECT SIR with the truth deformation field *φ*_0_ and perfect interpolation mapping *W* ^0^. Note that the stationary point is achieved only when the first-order necessary condition is satisfied for (13). Then, *C* ^*f*^ satisfies (see the proof in the appendix)

\begin{eqnarray*}\begin{array}{l}{\tilde{W}}_{{\varphi }_{0}}^{0}{H}^{\dagger }{P}_{L}({d}_{L},{W}_{{\varphi }_{0}}^{0}{C}^{f})+{H}^{\dagger }{P}_{H}({d}_{H},{C}^{f})+\lambda {\mathrm{\nabla }}{ \mathcal R }({C}^{f})\\ \quad =\,{\tilde{W}}_{{\varphi }_{0}}^{0}{H}^{\dagger }{S}_{L}({W}_{{\varphi }_{0}}^{0}{C}^{f})+{H}^{\dagger }{S}_{H}({C}^{f}).\end{array}\end{eqnarray*}

Let ${W}_{\varphi }={W}_{{\varphi }_{0}}^{0}+{\mathrm{\Delta }}W$ be the interpolation operator based on the estimated deformation field *φ*_*e*_, and Δ*W* be the difference between the estimated interpolation and the ‘truth’ interpolation, then the estimated image $\hat{C}$ satisfies

\begin{eqnarray*}{\tilde{W}}_{\varphi }{H}^{\dagger }{P}_{L}({d}_{L},{W}_{\varphi }\hat{C})+{H}^{\dagger }{P}_{H}({d}_{H},\hat{C})+\lambda {\mathrm{\nabla }}R({C}^{f})={\tilde{W}}_{\varphi }{H}^{\dagger }{S}_{L}({W}_{\varphi }\hat{C})+{H}^{\dagger }{S}_{H}(\hat{C}).\end{eqnarray*}

One could expand (21) at *C* ^*f*^ and ${W}_{{\varphi }_{0}}^{0}$, combining with (20) yields

\begin{eqnarray*}{\mathrm{\Delta }}C\approx -{\left({\tilde{W}}_{{\varphi }_{0}}^{0}{H}^{\dagger }{{\mathrm{\Lambda }}}_{L}{{HW}}_{{\varphi }_{0}}^{0}+{H}^{\dagger }{{\mathrm{\Lambda }}}_{H}H+\lambda {\mathrm{\Psi }}\right)}^{-1}\left({\mathrm{\Omega }}({\mathrm{\Delta }}W){HQ}-\tilde{W}{H}^{\dagger }{{\mathrm{\Lambda }}}_{L}H{\mathrm{\Delta }}{WC}\right),\end{eqnarray*}

where Λ_*L*_, Λ_*H*_, Ω(Δ*W*) and *Q* are defined in the appendix.

Equation (22) describes a linear relationship between the small perturbation of the deformation field Δ*W* and the change in the estimated image Δ*C*. The first two terms in (22) give the Fisher Information Matrix of the loss function, which provides a manifold on the domain of *c* describing the mean of gained information over all the random realizations of the observed data. Intuitively, an uniform image will not propagate any error to the result even if the DVF residual is large. The deformation error is propagated through the application on the image *C*. Small components in the inverted matrix will amplify the error introduced by estimated DVF. However, increasing the penalty strength *λ* helps reduce the error.

In order to ensure that maximum accuracy is achieved relative to data quality, a plausible requirement is that estimated image errors due to DVF errors are small compared to the errors caused by Poisson noise in the transmission data (Qi and Huesman 2004), i.e.

\begin{eqnarray*}{\mathbb{E}}[{\mathrm{\Delta }}C{\mathrm{\Delta }}{C}^{\dagger }]\prec \eta {{\mathrm{\Sigma }}}_{d},\end{eqnarray*}

where ${\mathbb{E}}$ denotes the expectation with respect to the random measurement, *η* is a tolerance factor (i.e. 0.01), and Σ_*d*_ denotes the covariance matrix caused by noise in data *d*. One can easily derive an approximation to Σ_*d*_ in the case of DECT (Fessler 1996), which is

\begin{eqnarray*}{\left(F+\lambda {\mathrm{\Psi }}\right)}^{-1}F{\left(F+\lambda {\mathrm{\Psi }}\right)}^{-1},\end{eqnarray*}

where $F={\tilde{W}}_{{\varphi }_{0}}^{0}{H}^{\dagger }{{\mathrm{\Lambda }}}_{L}{{HW}}_{{\varphi }_{0}}^{0}+{H}^{\dagger }{{\mathrm{\Lambda }}}_{H}H$.

Combining (23) and (24) yields

\begin{eqnarray*}{\mathbb{E}}\left({\tilde{W}}_{{\varphi }_{0}}^{0}{H}^{\dagger }{{\mathrm{\Lambda }}}_{L}H{\mathrm{\Delta }}{WC}{\left({\tilde{W}}_{{\varphi }_{0}}^{0}{H}^{\dagger }{{\mathrm{\Lambda }}}_{L}H{\mathrm{\Delta }}{WC}\right)}^{\dagger }\right)\prec \eta ({\tilde{W}}_{{\varphi }_{0}}^{0}{H}^{\dagger }{{\mathrm{\Lambda }}}_{L}{{HW}}_{{\varphi }_{0}}^{0}+{H}^{\dagger }{{\mathrm{\Lambda }}}_{H}H).\end{eqnarray*}

As it was mentioned in Qi and Huesman (2004), the comparison of traces of the matrices could be a surrogate of (25) for the sake of computational simplicity, i.e.

\begin{eqnarray*}\begin{array}{l}{\mathbb{E}}\left({\left({\tilde{W}}_{{\varphi }_{0}}^{0}{H}^{\dagger }{{\mathrm{\Lambda }}}_{L}H{\mathrm{\Delta }}{WC}\right)}^{\dagger }{\tilde{W}}_{{\varphi }_{0}}^{0}{H}^{\dagger }{{\mathrm{\Lambda }}}_{L}H{\mathrm{\Delta }}{WC}\right)\lt {tr}\left(\eta ({\tilde{W}}_{{\varphi }_{0}}^{0}{H}^{\dagger }{{\mathrm{\Lambda }}}_{L}{{HW}}_{{\varphi }_{0}}^{0}+{H}^{\dagger }{{\mathrm{\Lambda }}}_{H}H)\right)\\ \quad \approx {{\mathbb{E}}}_{\{| | v| | =1\}}\left(\eta v({\tilde{W}}_{{\varphi }_{0}}^{0}{H}^{\dagger }{{\mathrm{\Lambda }}}_{L}{{HW}}_{{\varphi }_{0}}^{0}+{H}^{\dagger }{{\mathrm{\Lambda }}}_{H}H){v}^{T}\right).\end{array}\end{eqnarray*}

### 2.5. Warped DEAM

In this section, we introduce the dual-energy alternating minimization (DEAM) algorithm as an example of DECT SIR. The DEAM algorithm is a statistical DECT algorithm that operates jointly on low and high-energy sinograms by decoupling the maximum log-likelihood problem at the energy domain using an alternating-minimizing strategy. The objective function of DEAM consists of the I-divergence and penalty term as

\begin{eqnarray*}\displaystyle \sum _{j}{ \mathcal I }({d}_{j}| | {g}_{j})+{ \mathcal R }(C),\end{eqnarray*}

where

\begin{eqnarray*}{ \mathcal I }({d}_{j}| | {g}_{j})=\displaystyle \sum _{y}{d}_{j}(y)\mathrm{log}\displaystyle \frac{{d}_{j}(y)}{{g}_{j}(y)}-{d}_{j}(y)+{g}_{j}(y).\end{eqnarray*}

Let *R* be the local regularization term that constrains the gradient of the image, such that

\begin{eqnarray*}{ \mathcal R }(C)=\displaystyle \sum _{x}\displaystyle \sum _{i}\displaystyle \sum _{x^{\prime} \in { \mathcal N }(x)}{w}_{R}(x,x^{\prime} )\psi \left({c}_{i}(x)-{c}_{i}(x^{\prime} )\right),\end{eqnarray*}

where ${ \mathcal N }(x)$ denotes the neighborhood of *x*, *w*_*R*_ denotes the weight addressing the distance between *x* and $x^{\prime} $, and the potential function *ψ* is a element-wise convex symmetric smooth function. The derivation of the image update with the penalty of the form (29) is shown in the appendix.

In DEAM, *ψ* is assumed to be a Huber-type potential function:

\begin{eqnarray*}\psi (t)={\delta }^{2}\left(\displaystyle \frac{| t| }{\delta }-\mathrm{ln}\left(1+\displaystyle \frac{| t| }{\delta }\right)\right),\end{eqnarray*}

where *δ* controls the transition between *ℓ* ^2^-norm and *ℓ* ^1^-norm. With an appropriate choice of *δ* (see appendix A.3 for the penalty parameter selection), this differentiable function preserves sparsity in the gradient domain. Then,

\begin{eqnarray*}{\left[{\mathrm{\nabla }}{ \mathcal R }({C}^{f})\right]}_{(i,x)}=2\displaystyle \sum _{x^{\prime} \in { \mathcal N }(x)}w(x,x^{\prime} ){\left.\displaystyle \frac{\partial \psi (\xi )}{\partial \xi }\right|}_{\xi ={c}_{i}^{f}(x)-{c}_{i}^{f}(x^{\prime} )}.\end{eqnarray*}

**Figure 3.**
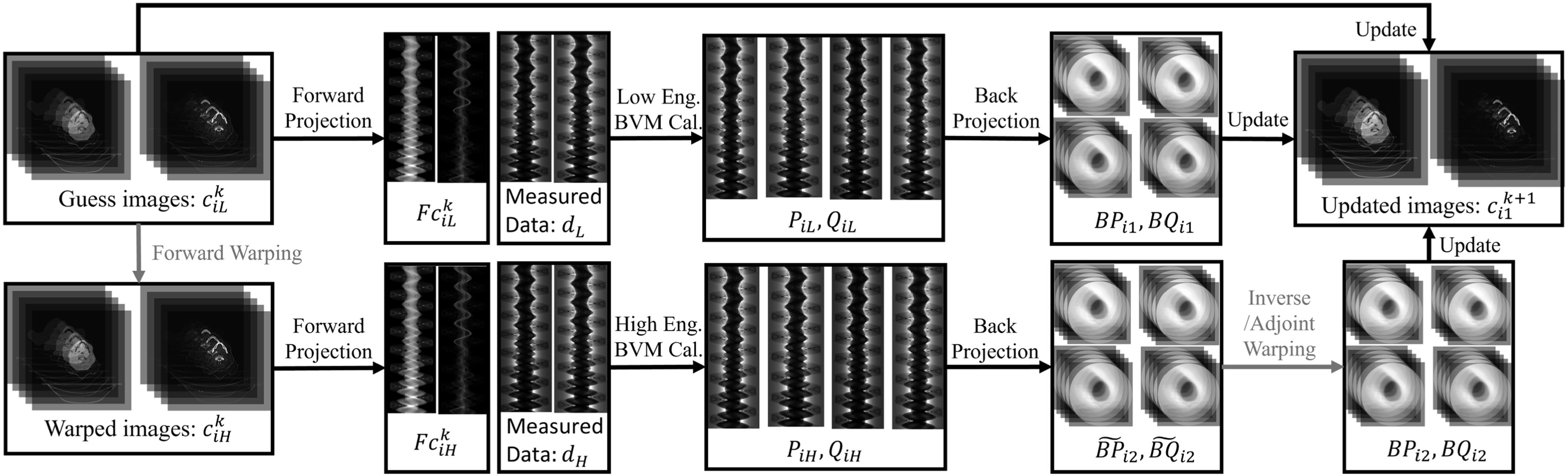
Flowchart of DEAM with registration.

When the high- and low-energy images are perfectly aligned, DEAM yields decomposed images that can estimate physical properties, e.g. charged-particle stopping powers and electron densities, with subpercentage uncertainties (Medrano *et al* 2022) on both synthetic and measured sinogram data acquired from clinical CT scanners (Medrano *et al* 2020). The flowchart of the modified version of DEAM, called warped DEAM, is shown in figure 3, and the algorithm is shown in algorithm 1.

Algorithm 1. DEAM with registration

**Table:** 

**while** Not Converged **do**
**for** *i* in $\{1,2\}$ **do** ▹*Inverse warping*
${c}_{i2}\leftarrow {c}_{i1}\circ {\varphi }^{-1}$
**for** $i,j$ in $\{1,2\}$ **do** ▹*Forward-projection*
${c}_{{ij}}^{F}(y)\leftarrow {\sum }_{x}h(x,y){c}_{{ij}}(x)$
**for** *j* in {1, 2} **do** ▹*Basis vector model*
${q}_{j}(y,E)\leftarrow {I}_{0,j}(y,E){e}^{-{\sum }_{i}{\mu }_{i}(E){c}_{{ij}}^{F}(y)}$
**for** $i,j$ in $\{1,2\}$ **do**
${\tilde{g}}_{{ij}}(y)\leftarrow {\sum }_{E}{\mu }_{i}(E){q}_{j}(y,E)$
${\tilde{d}}_{{ij}}(y)\leftarrow \tfrac{{\sum }_{E}{\mu }_{i}(E){q}_{j}(y,E){d}_{j}(y)}{{\sum }_{E^{\prime} }{q}_{j}(y,E^{\prime} )}$
**for** $i,j$ in $\{1,2\}$ **do** ▹*Back-projection*
${g}_{{ij}}^{B}(x)\leftarrow {\sum }_{y}h(x,y){\tilde{g}}_{{ij}}(y)$
${d}_{{ij}}^{B}(x)\leftarrow {\sum }_{y}h(x,y){\tilde{d}}_{{ij}}(y)$
**for** *i* in $\{1,2\}$ **do** ▹*Forward warping*
${g}_{i2}^{B}(x)\leftarrow {g}_{i2}^{B}(x)\circ \varphi $
${d}_{i2}^{B}(x)\leftarrow {d}_{i2}^{B}(x)\circ \varphi $
**Solve decoupled function** ▹*Image update*
${\mathrm{argmin}}_{{c}_{i1}}{\sum }_{j}{\sum }_{i}{\sum }_{x}\left[{d}_{{ij}}^{B}(x){c}_{i1}^{{new}}+{g}_{{ij}}^{B}(x)\tfrac{1}{{Z}_{i}(x)}\cdot \right.$
$\left.\exp ({Z}_{i}(x)[{c}_{i1}^{{old}}(x)-{c}_{i1}^{{new}}(x)])\right]+R({{\bf{c}}}^{k+1})$
**outputs:**
${c}_{1}={c}_{11}$, ${c}_{2}={c}_{21}$

In this work, the warped DEAM algorithm is accelerated by the ordered subset technique (Erdogan and Fessler 1999) during the early iterations. The GPU-accelerated code has been implemented so that the algorithm is run on 4 NVIDIA V100 for time efficiency.

### 2.6. Deformable registration

In our setting, we require a DIR algorithm that yields diffeomorphic, topology-preserving mappings.

Since we need to repetitively and reciprocally warp image volumes between two different material energy domains (*c*_1_ and *c*_2_), a symmetric algorithm that guarantees the invertibility of the estimated transformation is critical to the whole reconstruction process. This guarantees the inverse consistency of the output mappings. Since the patient’s anatomy may vary during two successive scans, the algorithm should also be stable with large-scale deformation. Of the algorithms meeting these specifications (Oliveira and Tavares 2014), the state-of-the-art advanced normalization tools (ANTs) (Avants *et al* 2009) toolkit was selected for our warped DEAM pipeline.

Designating the 90 and 140 kVp images as the fixed image *J* and moving image *I*. The ANTs DIR algorithm (symmetric image normalization method (SyN) (Avants *et al* 2008)) computes the optimal transformation *φ* ^*^ within a transformation space that maps the coordinates ***x*** ∈ Ω of the moving image *I*(***x***) to a location ${\boldsymbol{x}}^{\prime} \in {\mathrm{\Omega }}$ which minimize a cost function *E* describing the similarity between *I* and *J*.

SyN generates transformations in a diffeomorphic space that forms a group of differentiable maps with differentiable inverses (Ebin and Marsden 1970) that is closed under composition. The diffeomorphism *φ* is defined on the image domain Ω and is parameterized over time as a family of diffeomorphisms, *φ*(***x***, *t*): Ω × *t* → Ω, *t* ∈ [0, 1], which can be calculated by integrating a time-dependent, smooth velocity field, $v:{\mathrm{\Omega }}\times t\to {{\mathbb{R}}}^{d}$, described by the following ordinary differential equation (ODE)

\begin{eqnarray*}\displaystyle \frac{d\varphi ({\boldsymbol{x}},t)}{{dt}}=v(\varphi ({\boldsymbol{x}},t),t),\varphi ({\boldsymbol{x}},0)={\boldsymbol{x}}.\end{eqnarray*}

The final DVF yielded by *φ* is **u**(***x***) = *φ*(***x***, 1) − ***x***. SyN derives the path by decomposing the final diffeomorphism *φ* into two symmetric components *φ*_1_ and *φ*_2_. Now define, in *t* ∈ [0, 0.5], *v*(***x***, *t*) = *v*_1_(***x***, *t*) and *v*(***x***, *t*) = *v*_2_(***x***, 1 − *t*) when *t* ∈ [0.5, 1]. This leads to an optimization problem as

\begin{eqnarray*}\begin{array}{rcl}\{{v}_{1}^{* },{v}_{2}^{* }\} &amp; = &amp; {\mathrm{argmin}}_{v}E={\mathrm{argmin}}_{v}\left\{{\int }_{0}^{0.5}{\parallel {{Lv}}_{1}({\boldsymbol{x}},t)\parallel }^{2}{dt}+{\int }_{0}^{0.5}{\parallel {{Lv}}_{2}({\boldsymbol{x}},t)\parallel }^{2}{dt}\right.\\ &amp; &amp; \left.+\lambda {\int }_{{\mathrm{\Omega }}}{ \mathcal M }\left(I\circ {\varphi }_{1}({\boldsymbol{x}},0.5),J\circ {\varphi }_{2}({\boldsymbol{x}},0.5)\right)d{\boldsymbol{x}}\right\},\end{array}\end{eqnarray*}

where ${ \mathcal M }$ is the similarity metric depending on the images and the transformation, *λ* is a parameter controlling the tradeoff between smoothness and image similarity, *L* is a differential operator that enforces smoothness of the vector field, and $\parallel {Lv}\parallel $ represents the vector norm of *v*.

Note that the regularization term in (33) is the total distance between the initial and the final transformations, which enforces a geodesic property on the resulting transformations. The resulting Euler–Lagrange equations are solved by variational optimization (Beg *et al* 2005).

To find the optimal *v* ^*^, the variational energy *E* is minimized from either endpoint towards the midpoint of the transformation, as indicated by the similarity term. This strategy ‘splits’ the optimization dependence equally between both images. Thus, gradient-based iterative convergence deforms *I* and *J* along the geodesic diffeomorphism, *φ*, to a fixed point midway (intuited by the notion of shape distance) motivating the moniker ‘symmetric normalization’ (SyN) for the solution strategy (Avants *et al* 2011).

As discussed in section 2.10, a multi-modality similarity term must be used since the attenuation coefficients of the same material differ significantly from 90 to 140 kVp.

### 2.7. Initialization

We use the iterative filtered back projection (iFBP) method to initialize DEAM (Yan *et al* 2000). iFBP is an image-domain decomposition method that incorporates the polychromatic characteristics of the x-ray source into reconstruction and iteratively updates the image by the FBP of the discrepancy between the monochromatic and the polychromatic sinograms. However, since the estimated image is iteratively projected for the sinogram evaluation in iFBP, the inverse DVF should be applied prior to each forward projection.

### 2.8. Simulation studies

**Figure 4.**
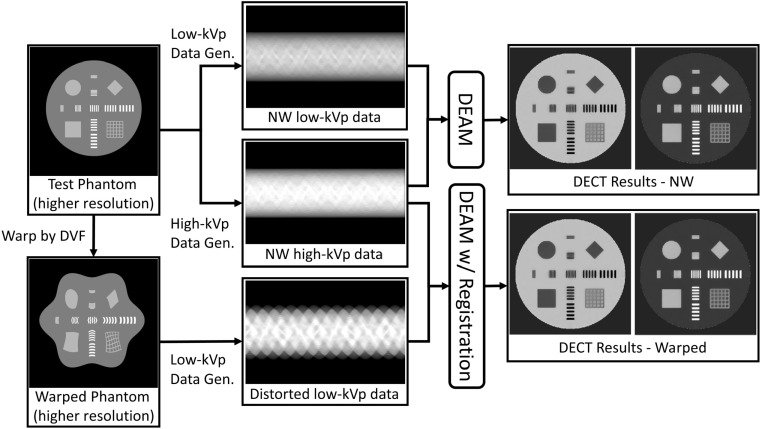
The flowchart of the simulation study on high-resolution phantom and illustration of harmonic DVF.

Two synthetic phantoms were used to evaluate the performance of the proposed motion-compensated DEAM method. The first was a modified high-contrast resolution phantom shown in figure 4 consisting of disks, squares, and grids at 1–5 intervals composed of 18% CaCl_2_ solution embedded in a 30 cm diameter water cylinder. The resolution insert bars were composed of 45% K_2_HPO_4_ solution. The flowchart for this test is shown in figure 4. The phantom was warped by a harmonic deformation field that has an exact analytical inverse. The original and warped phantoms were then back-projected to generate the non-warped (NW) and distorted geometry synthetic transmission data.

The dimensionality and voxel size of the phantom were 2440 × 2440 × 52 and 0.25 × 0.25 × 1 mm, respectively, so as to preserve its sub-millimeter information. The phantom is warped by a harmonic deformation field that has a strict analytical inverse. Then, the original DEAM and warped DEAM algorithms jointly operated on the low- and high-energy synthetic sinograms for the non-warped and warped phantom geometries.

For the anthropomorphic simulation study, we utilize the advanced version of the 4D digital phantom 4D Extended Cardiac-Torso (XCAT) (Segars *et al* 2010) as the ground truth. XCAT is an anthropomorphic phantom based on a non-uniform rational B-spline (NURBS) with highly detailed whole-body anatomies. The deformation fields are given by parameterized motion models with the heart and respiratory cycles. The XCAT phantom CT projector generates projections via continuous line integrals from the NURBS file rather than the discretized phantom. Synthetic polychromatic CT sinograms were constructed using the continuous XCAT projector in fan beam mode. We used a script to simulate the helical scanning process that duplicated Philips Big Bore scanner geometry and scan settings. The incident photon-fluence profile was scaled so that the variance of XCAT image reconstructed by the in-house FBP algorithm matched the variance of the clinical FBP image reconstructed by the same algorithm.

### 2.9. Clinical study

Helical 90 and 140 kVp scans of a patient subject (IRB study NCT03403361) were sequentially acquired on a 16-row Philips Brilliance Big Bore CT scanner with 0.75 mm × 16 row (12 mm) collimation. The scanned region extended from the top of the head to the base of the skull to model a typical CNS patient and from the base of the skull to the top of the pelvis to represent a typical lung patient. The central-axis spectra used in the reconstruction process were experimentally determined by fitting the well-established Birch Marshall model to measured transmission profiles through aluminum and copper attenuators of varying thickness (Evans *et al* 2013).

The raw sinograms were exported directly from the scanner and preprocessed without beam hardening correction using proprietary software provided by the vendor. The sinograms were then reconstructed with our original and warped DEAM with a resolution of 1 × 1 × 1.034 mm^3^. The *z*-direction resolution was chosen to ensure that eight slices covered exactly a single gantry rotation. A research-oriented version of DEAM algorithm was run for 400 iterations with 33 ordered subsets followed by 500 iterations without ordered subsets. Each reconstruction took approximately 8 h for the component weights with a size of 610 × 610 × 146.

### 2.10. Similarity and image quality metrics

In this work we use two metrics, mutual information (MI) and local cross-correlation (LCC), for multispectral image registration.

MI quantifies the similarity between two images by predictability, which is the information gained about one image by observing the other. Let *P*_*I*_(*a*) denote the probability that value *a* appears in image *I*, *P*_*J*_(*b*) denote the probability that value *b* appears in image *J*, and *P*_*IJ*_(*a*, *b*) denote the joint probability that value *a* appears in image *I* and value *b* appears in image *J*. Then,

\begin{eqnarray*}{MI}(I;J)=\displaystyle \sum _{a}\displaystyle \sum _{b}{p}_{{IJ}}(a,b)\mathrm{log}\left(\displaystyle \frac{{p}_{{IJ}}\left(a,b\right)}{{p}_{I}\left(a\right){p}_{J}\left(b\right)}\right)\end{eqnarray*}

LCC quantifies the similarity using the local Pearson correlation coefficient.

\begin{eqnarray*}\bar{I}(x)=I(x)-\displaystyle \frac{1}{| N(x)| }\displaystyle \sum _{x^{\prime} \in N(x)}I(x^{\prime} )\end{eqnarray*}

\begin{eqnarray*}\bar{J}(x)=J(x)-\displaystyle \frac{1}{\left|\left.N(x\right)\right|}\sum _{\left.{x}^{{\mathrm{{\prime} }}}\in N(x\right)}J({x}^{{\mathrm{{\prime} }}})\end{eqnarray*}

\begin{eqnarray*}\mathrm{LCC}(I,J,x)=\displaystyle \frac{{\left({\sum }_{x^{\prime} \in N(x)}\bar{I}(x^{\prime} )\bar{J}(x^{\prime} )\right)}^{2}}{\left({\sum }_{x^{\prime} \in N(x)}\bar{I}{\left(x^{\prime} \right)}^{2}\right)\left({\sum }_{x^{\prime} \in N(x)}\bar{J}{\left(x^{\prime} \right)}^{2}\right)}.\end{eqnarray*}

The point-wise mutual information (PMI) is utilized as the misalignment indicator of two images to visualize the registration improvement (Rogelj *et al* 2003):

\begin{eqnarray*}{\mathrm{PMI}}_{{IJ}}(x)={p}_{{IJ}}\left(I(x),J(x)\right)\cdot \mathrm{log}\left(\displaystyle \frac{{p}_{{IJ}}\left(I(x),J(x)\right)}{{p}_{I}\left(I(x)\right){p}_{J}\left(J(x)\right)}\right).\end{eqnarray*}

In order to quantitatively assess the performance of the proposed motion-compensation framework with DEAM, relative LAC bias and mean absolute error (MAE) are introduced against the reference value at different energies

\begin{eqnarray*}\mathrm{bias}(E)=\displaystyle \frac{1}{N}\displaystyle \sum _{x}\displaystyle \frac{{c}_{1}(x){\mu }_{1}(E)+{c}_{2}(x){\mu }_{2}(E)-{\mu }_{{ref}}(E)}{{\mu }_{{ref}}(E)}\end{eqnarray*}

and

\begin{eqnarray*}\mathrm{MAE}(E)=\displaystyle \frac{1}{N}\displaystyle \sum _{x}\displaystyle \frac{| {c}_{1}(x){\mu }_{1}(E)+{c}_{2}(x){\mu }_{2}(E)-{\mu }_{{ref}}(E)| }{{\mu }_{{ref}}(E)},\end{eqnarray*}

respectively, where *μ*_*ref*_ denote the reference LACs of the selected tissue.

In addition, we also use the SPR estimation error as another metric to assess the performance of the proposed method. The stopping power mappings are estimated from DEAM basis component images following the procedure outlined in previous studies [41], and the estimated SPR is compared to the ground truth value derived from known material composition with respect to the bias and the mean absolute error.

## 3. Results

### 3.1. Resolution simulation results

**Figure 5.**
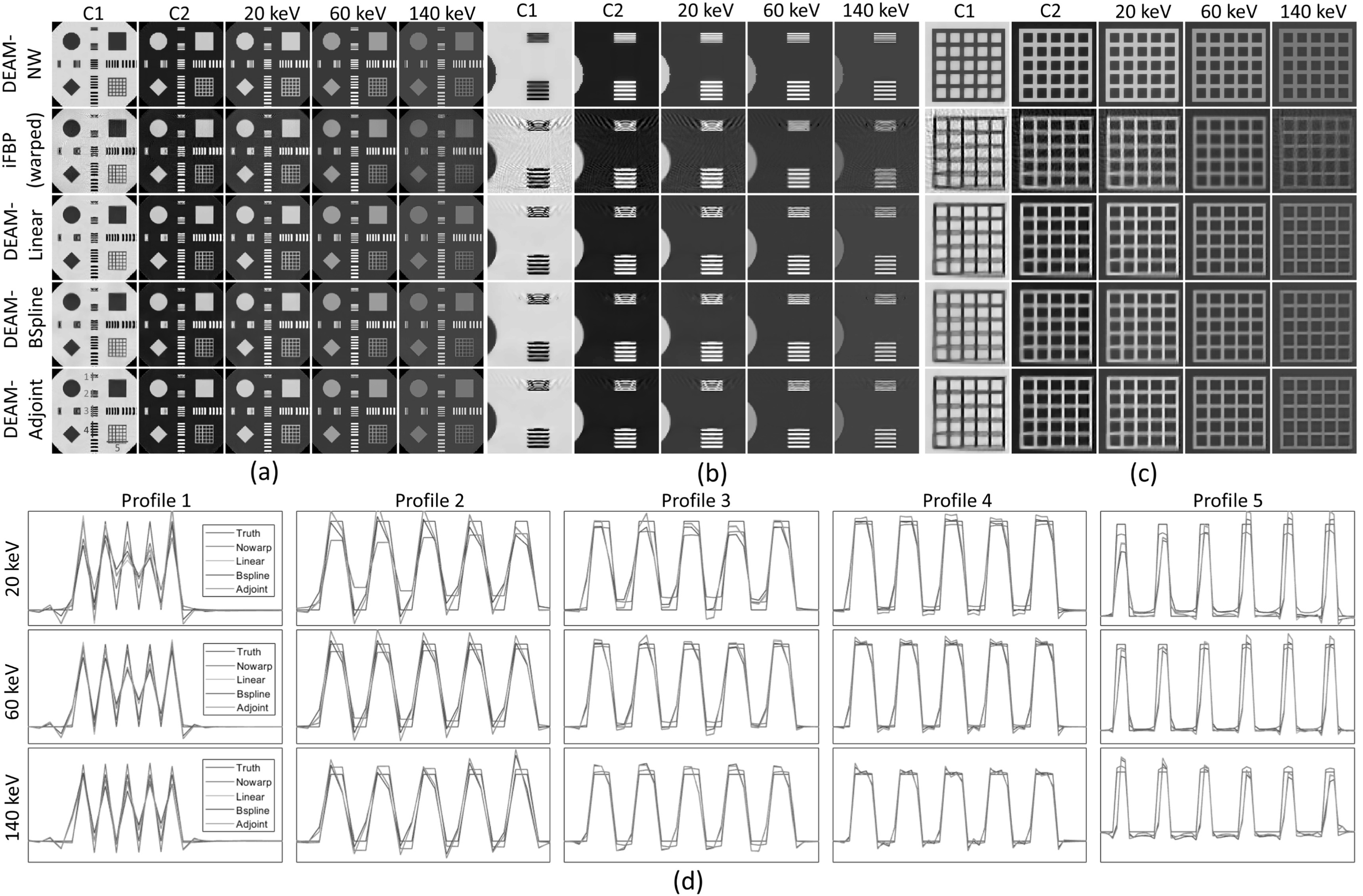
(a) The reconstructed images. From the top row to the bottom: DEAM-NW: baseline (reconstruction from undistorted sinograms), iFBP: initial condition (image-based decomposition with image warping), DEAM-Linear: warped DEAM with linear interpolation (Jacobian-weighted inverse), DEAM-Bspline: warped DEAM with B-Spline interpolation (Jacobian-weighted inverse), and DEAM-adjoint: warped DEAM with linear interpolation (adjoint). From left column to the right: *c*_1_ with display window [–0.2, 1.2], *c*_2_ with display window [–0.2, 1.2], virtual mono-energetic image (VMI) at 20, 60, and 140 keV estimated from *c*_1_ and *c*2_._ (b) and (c): zoomed-in version of the reconstructed images. (d) Shows the profiles of the different DEAM algorithms through five regions of interest (ROI) shown in the lower-left image of (a).

The reconstructed images for the high-contrast study are shown in figure 5(a). Figures 5(b) and (c) show the zoomed details of the corresponding reconstruction. As expected, the DEAM-NW image has the cleanest edges, and the iFBP image has the most severe artifact, especially around the upper region in 5(b) and left region in 5(c). DEAM-Linear (DEAM-LI), DEAM-BSpine (DEAM-BS), and DEAM-Adjoint (DEAM-AD) share a similar pattern of the artifact in the region of 1 mm spacing bars, and DEAM-BS performs better than DEAM-AD and DEAM-LI. The lower left corn of the reconstructed grids in 5(c) are blurred, and the DEAM-LI and DEAM-AD images are slightly better than DEAM-BS. Moreover, with the increase of the energy for VMIs, the artifact would be reduced. Figure 5(d) shows profiles through the various bar patterns. Since the warping magnitude varies across the bar pattern, the profiles are not identical for bars within the same group. In profile 1, the outer and inner bars are over- and under-estimated, respectively, by the three warped DEAM algorithms. As bar spacing increases, the differences between bars in the same spacing group are reduced. For the 4 mm pattern, the warped DEAM profiles closely approximate those of the non-warped DEAM image.

Regarding the dependence of image quality on DEAM interpolation scheme, none standards out as unambiguously superior. For example, in profile 1, DEAM-BS and DEAM-AD outperform DEAM-LI at 20 keV, whereas DEAM-LI and DEAM-AD outperform DEAM-BS at 140 keV. In profile 3, DEAM-LI and DEAM-AD outperform DEAM-BS at 20 keV, but in profile 4, DEAM-BS outperforms DEAM-LI and DEAM-AD at 20 keV. Considering both the overall performance and the computational complexity, DEAM with adjoint was used in later studies.

### 3.2. Anthropomorphic simulation results

**Figure 6.**
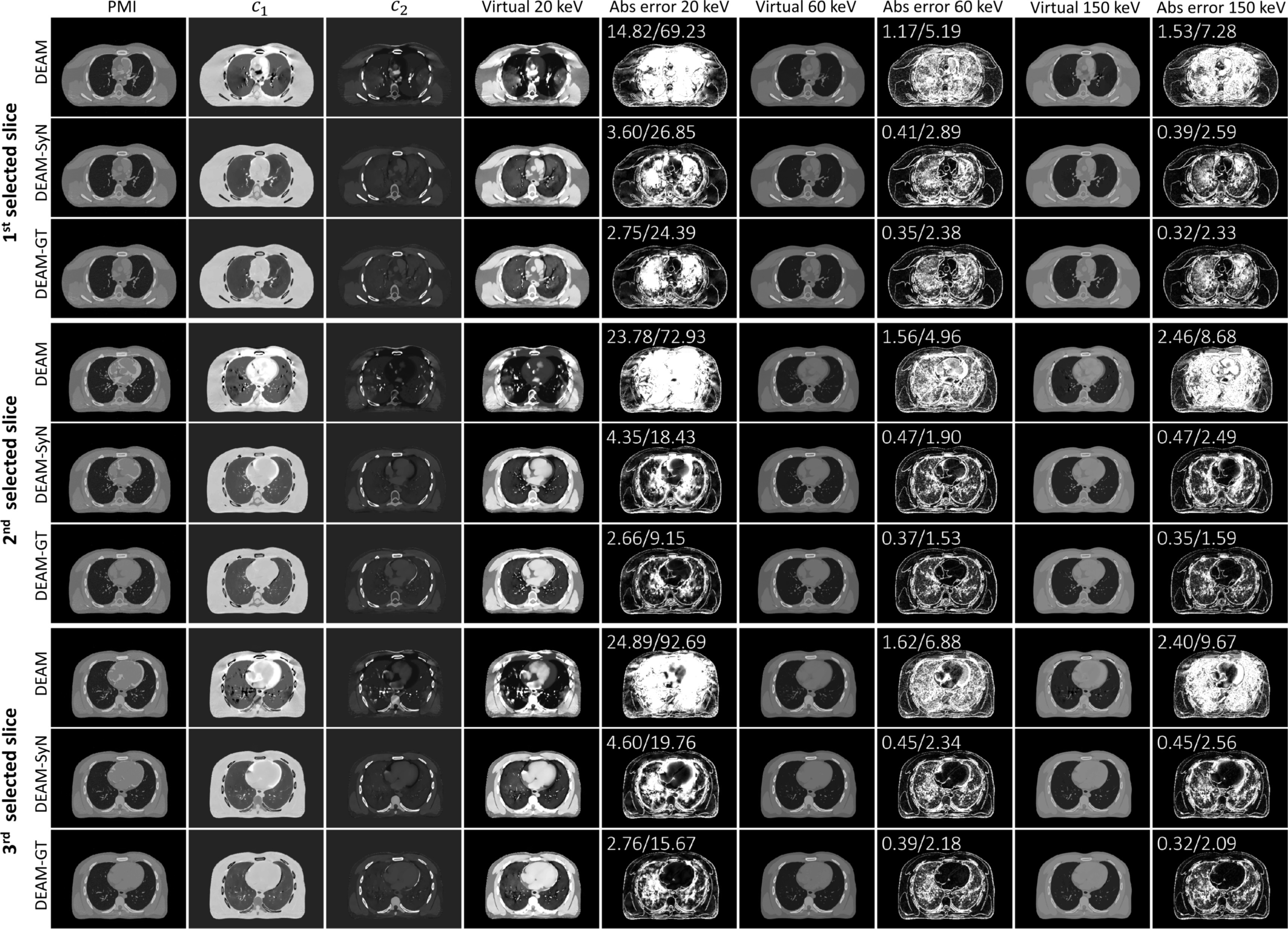
Three different slices from DEAM reconstructions of XCAT phantom without registration (DEAM), motion-compensated DEAM (DEAM-SyN), and motion-compensated DEAM using the XCAT ground truth DVF (DEAM-GT). From left to right the columns show PMI overlaid on the 140 kVp image, *c*_1_ image (display window [–0.2, 1.2]), *c*_2_ image (display window [–0.2, 1.2]), 20 keV VMI with the display window [0, 0.1], percentage absolute error to the ground truth at 20 keV with the display window [0%, 30%], 60 keV VMI from *c*_1_ and *c*_2_ with the display window [0, 0.05], percentage absolute error to the ground truth at 60 keV with the display window [0%, 5%], and 150 keV VMI from *c*_1_ and *c*_2_ with the display window [0, 0.03], percentage absolute error to the ground truth at 150 keV with the display window [0%, 5%]. The numbers in the upper left corner of each error image denote the percentage MAE for extra-lung soft-bony tissue and lung parenchyma, respectively.

Figure 6 shows the performance of DEAM with different deformation fields on three different slices of the XCAT phantom. The PMI images demonstrate the pointwise similarity between 140-kVp fixed image and deformed 90-kVp image.

DEAM-SyN and DEAM-GT both outperform DEAM. The percentage MAEs in the region of the soft tissues and spine for DEAM-SyN are reduced by 12.55, 3.27, and 4.88-fold compared to the result without registration at 20, 60, and 150 keV, respectively.

For extra-lung tissue, incorporating warped DEAM using GT reduces MAEs from 14.82%–24.89% to 2.66%–2.76% for 20 keV LAC For 150 keV, warped DEAM with GT DVF reduces the errors from 1.53%–2.46% to 0.32%–0.35%. For lung parenchyma, the corresponding error reductions are 44.84%–77.02% for 20 keV LAC, and 4.95%–7.58% for 150 keV LAC The LAC of lung parenchyma is small compared to soft and bony tissues, leading to the small denominator in MAE. Therefore, MAEs for lung parenchyma are significantly larger than MAEs for extra-lung tissue.

DEAM-SyN errors are only modestly higher than DEAM-GT errors. It could be seen that the mismatches in PMI images and artifacts in reconstructed images are mainly concentrated in the heart region. The artifacts caused by misalignment match the shape of red regions in PMI. Most of the mismatching artifacts are corrected by the DVF from SyN. However, DEAM-GT still outperforms DEAM-SyN in reconstructing the boundaries of organs.

**Figure 7.**
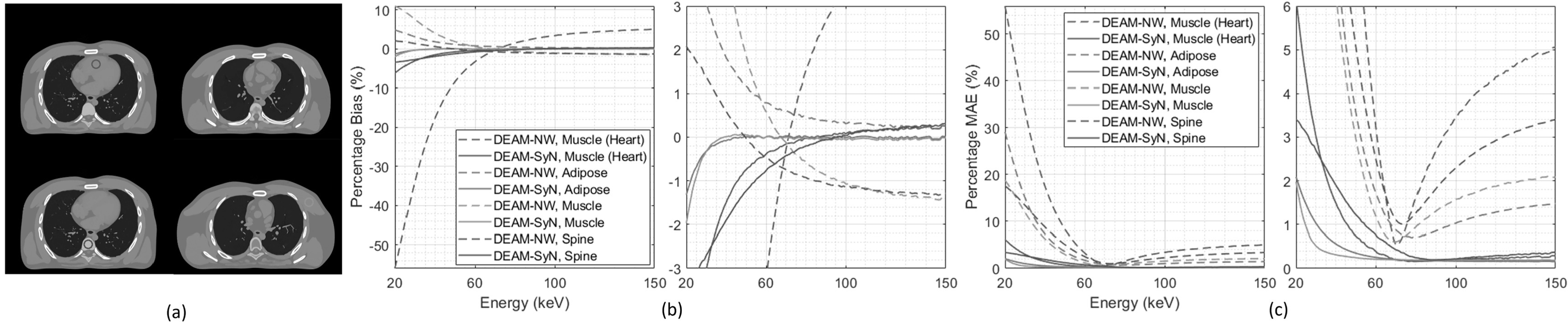
(a) Four regions of interest in the XCAT phantom indicated by circles. Plots and the zoomed plots of (b) percentage bias and (c) absolute error versus energy for original and warped DEAM reconstructions of monoenergetic LACs of the XCAT phantom.

Figure 7 shows the accuracy with which DEAM XCAT reconstructions with and without motion compensation estimate mono-energetic linear attenuation coefficients in muscle (heart), adipose, muscle, and spine, respectively. The selected ROIs are 10 mm long cylinders. Figure 7(b) and (c) shows that motion compensation significantly reduces the magnitude of bias and MAE. For example, bias for heart muscle is reduced from 56% to 8% and 5% to 0.4% at 20 keV, 150 keV, respectively. The same trend can also be seen in other regions. The magnitude of bias for adipose and muscle is reduced from 1.7% and 4% to 0.2% and 0.1% at 40 keV, respectively. Both the bias and MAE for warped DEAM in the selected regions are within 1% from 40 to 150 keV, except for the heart muscle. The magnitude of bias and MAE for heart muscle is within 1% after 55 keV.

**Figure 8.**
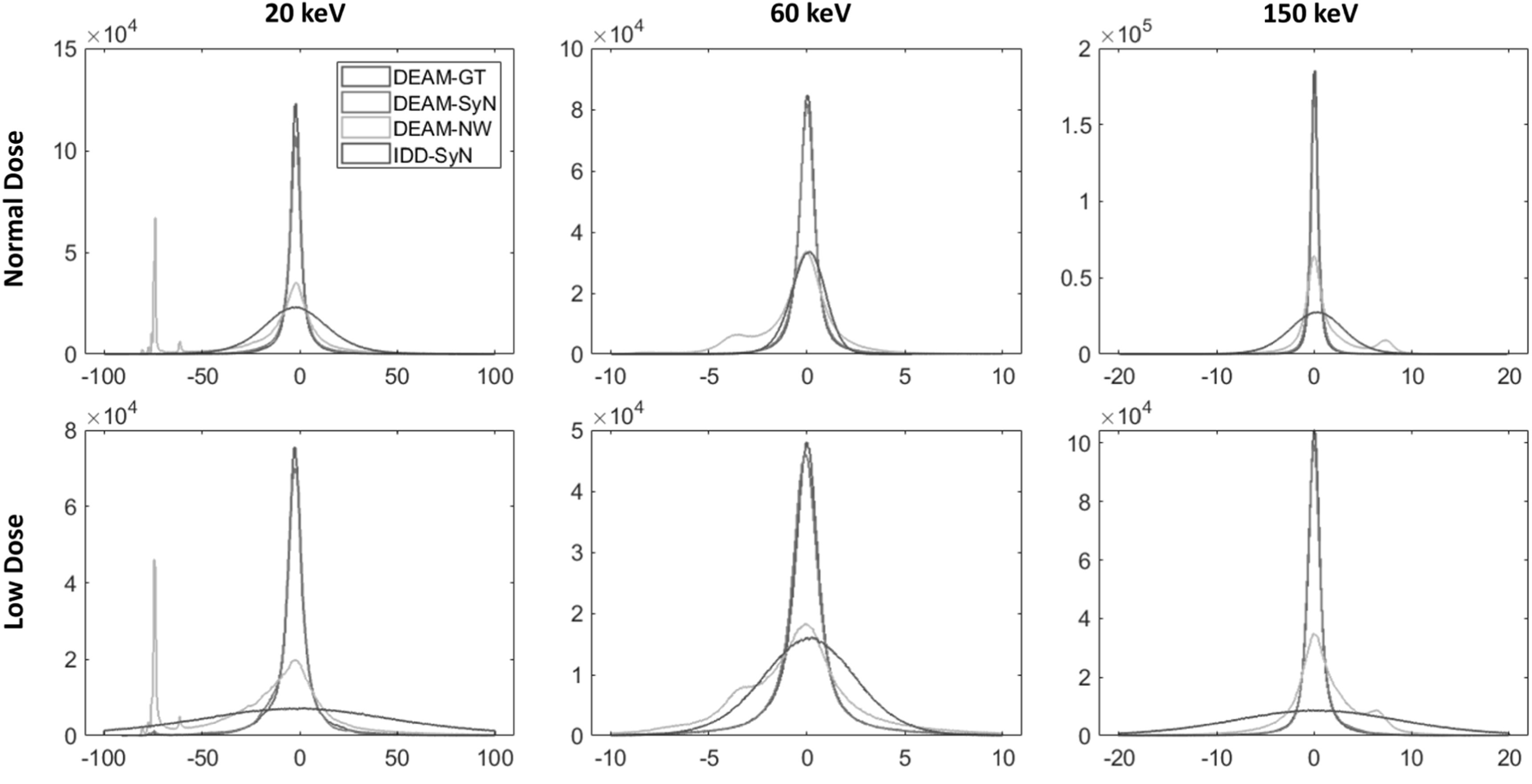
Histograms of percentage errors of LACs reconstructed by different DEAM algorithms against the ground truth (*μ*_*GT*_(*x*, *E*) − *μ*_*est*_(*x*, *E*)). A higher concentration of values near zero indicates better performance. The distribution tails were truncated to enhance clarity and legibility. The columns from left to right correspond to the estimated LAC at different energies. The first and second rows illustrate the performance of the normal dose and low dose simulation studies, respectively. IDD-SyN denotes the image-domain decomposition results with SyN-estimated DVF. *X*-axis unit: %.

To assess the robustness of the proposed pipeline against noise, we conducted a quantitative comparison between the results reconstructed from the simulated normal-dose and low-dose measurements of the XCAT phantom, where the low-dose measurement was simulated at 1/10 of the normal dose. In order to highlight the superiority of our approach, we conducted a performance comparison between our proposed method and the pure image-domain decomposition method that is widely used in clinical practice. In image domain decomposition, two bead-hardening-corrected measurements were reconstructed by the filtered back projection, and the low-kVp image was warped by the estimated DVF. Subsequently, two basis component images are obtained by linearly combining the low- and high-kVp images, which we refer to as IDD-SyN. The results are presented in figure 8. Each histogram plots the pointwise errors between the estimated LAC and the ground truth LAC, divided by the ground truth LAC, in a 610 × 610 × 100 image volume. We only considered non-boundary voxels corresponding to soft and bony tissues in this assessment to avoid extremely small values being used as the denominator.

The evaluation results indicate that both DEAM-GT and DEAM-SyN algorithms reconstruct the results with negligible bias and variance in the normal dose and low dose studies. The majority of the errors fall within the range of −1% to 1% at 60 and 150 keV for normal dose. The histograms of DEAM-NW at 60 and 150 keV exhibit two peaks, where one peak represents the correctly reconstructed values, and the other denotes the values influenced by the misalignment. In the 20 keV results, the peak corresponding to the ‘correct’ values is greatly reduced, indicating that motion artifacts in DECT SIR can significantly affect the fixed region even when the target energy is relatively low.

When comparing DEAM-SyN with IDD-SyN in the normal-dose case, we found that the FWHM of the IDD-SyN histogram is approximately 1.8 times larger than that of the DEAM-SyN histogram at 60 keV. This difference becomes more pronounced for other energies. Specifically, the FWHM for DEAM-SyN is around 11.7 and 8.9 times larger than the FWHM for IDD-SyN at 20 and 150 keV, respectively. Additionally, the estimation biases of IDD-SyN are larger than those of DEAM-SyN. As a result, our study demonstrates that the image-domain decomposition method with the estimated DVF leads to greater bias and variance compared to the proposed algorithm with the same DVF.

**Figure 9.**
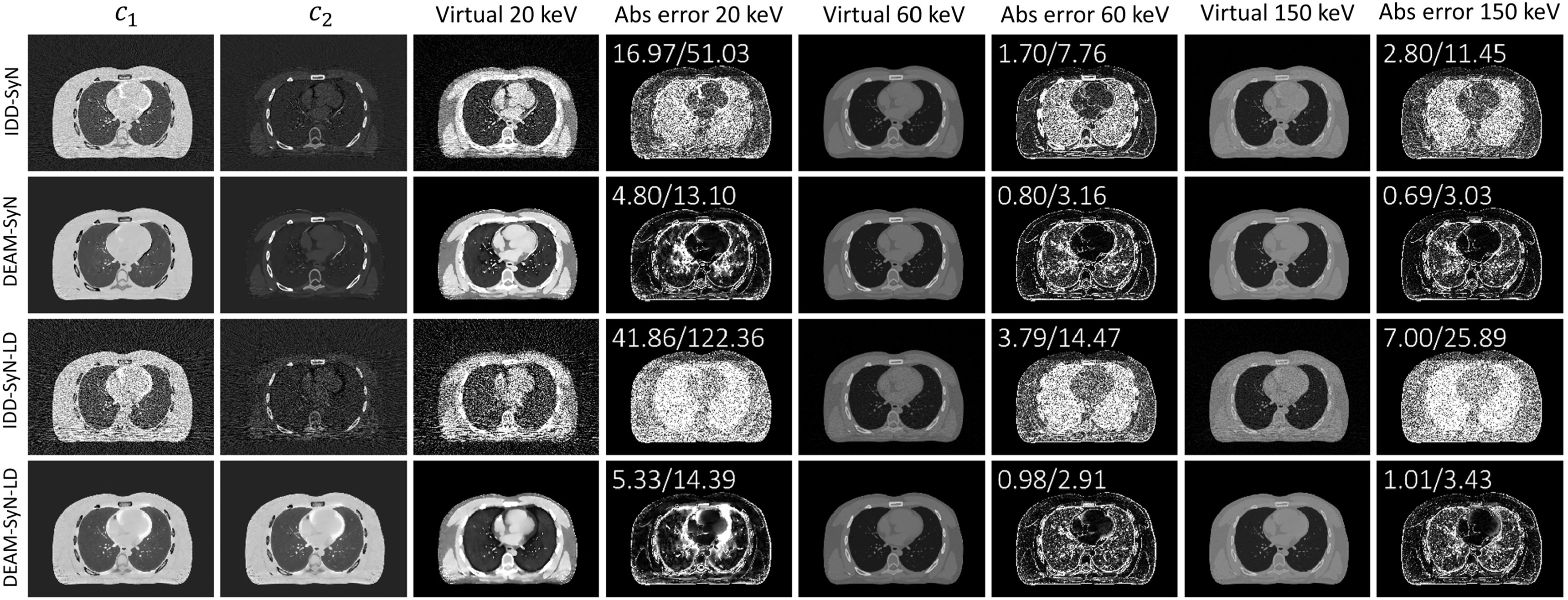
A representative slice of the reconstructed image. IDD-SyN and DEAM-SyN denote the reconstructed image from the normal dose measurement, and IDD-SyN-LD and DEAM-SyN-LD denote the reconstructed image from the low dose measurement. The numbers in the upper left corner of each error image denote the percentage MAE for extra-lung soft-bony tissue and lung parenchyma, respectively.

**Figure 10.**
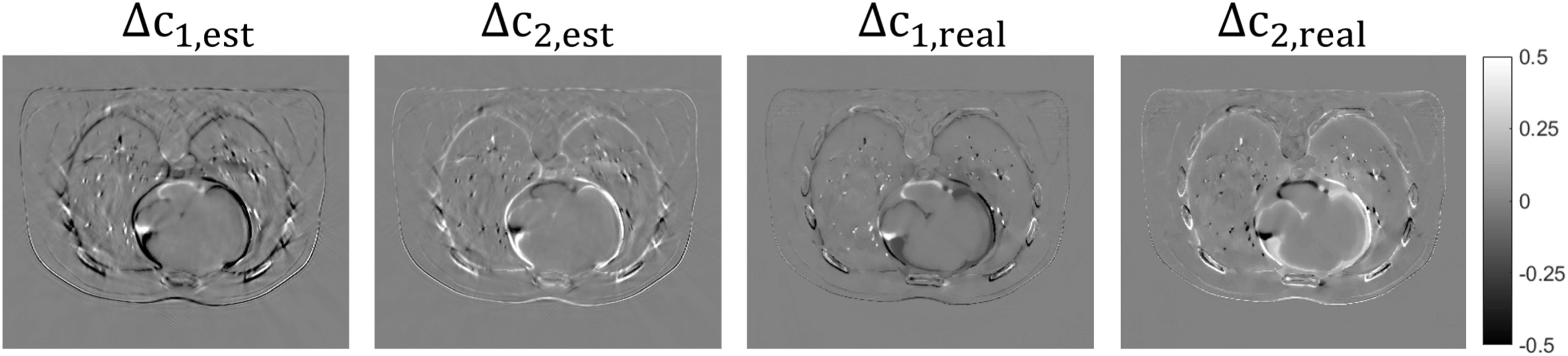
Theoretical and practical errors introduced by inaccurate DVF.

Moreover, since the penalty weight in DECT SIR can be adjusted to compensate for the noise, our proposed method is less likely to be affected by variance in the measurement than the image-domain decomposition method. In the low dose case, we multiply the penalty weight by a factor of 3 to balance the noise level and resolution of the reconstructed result. The FWHM of low-dose DEAM-SyN errors is approximately 1.6 times larger than that FWHM in the normal-dose case at 60 keV, while the FWHM of low-dose IDD-SyN errors is approximately 3.1 times larger than that at 60 keV. Figure 9 depicts a representative slice of the reconstructed image. As the simulated dose decreases, the image reconstructed by IDD exhibits a notable increase in noise levels, while DEAM-SyN with the appropriate penalty weight effectively suppresses the noise. However, DEAM-SyN-LD exhibits a larger boundary error in comparison to DEAM-SyN, especially at 20 keV. This discrepancy is likely due to the intensified penalty strength employed in DEAM-SyN-LD. Figure 10 shows propagated uncertainty in BVM component weights due to DVF inaccuracies, where Δ*c*_est_ is calculated from our first-order theoretical analysis, equation (22), and Δ*c*_real_ denotes the actual observed difference between warpred DEAM reconstructions based on-SyN and ground-truth DVFs. Note that SyN registration errors are sufficiently large that the inequality (26) is not satisfied (the error mostly clustered around the organ boundaries.)

### 3.3. Clinical results

**Figure 11.**
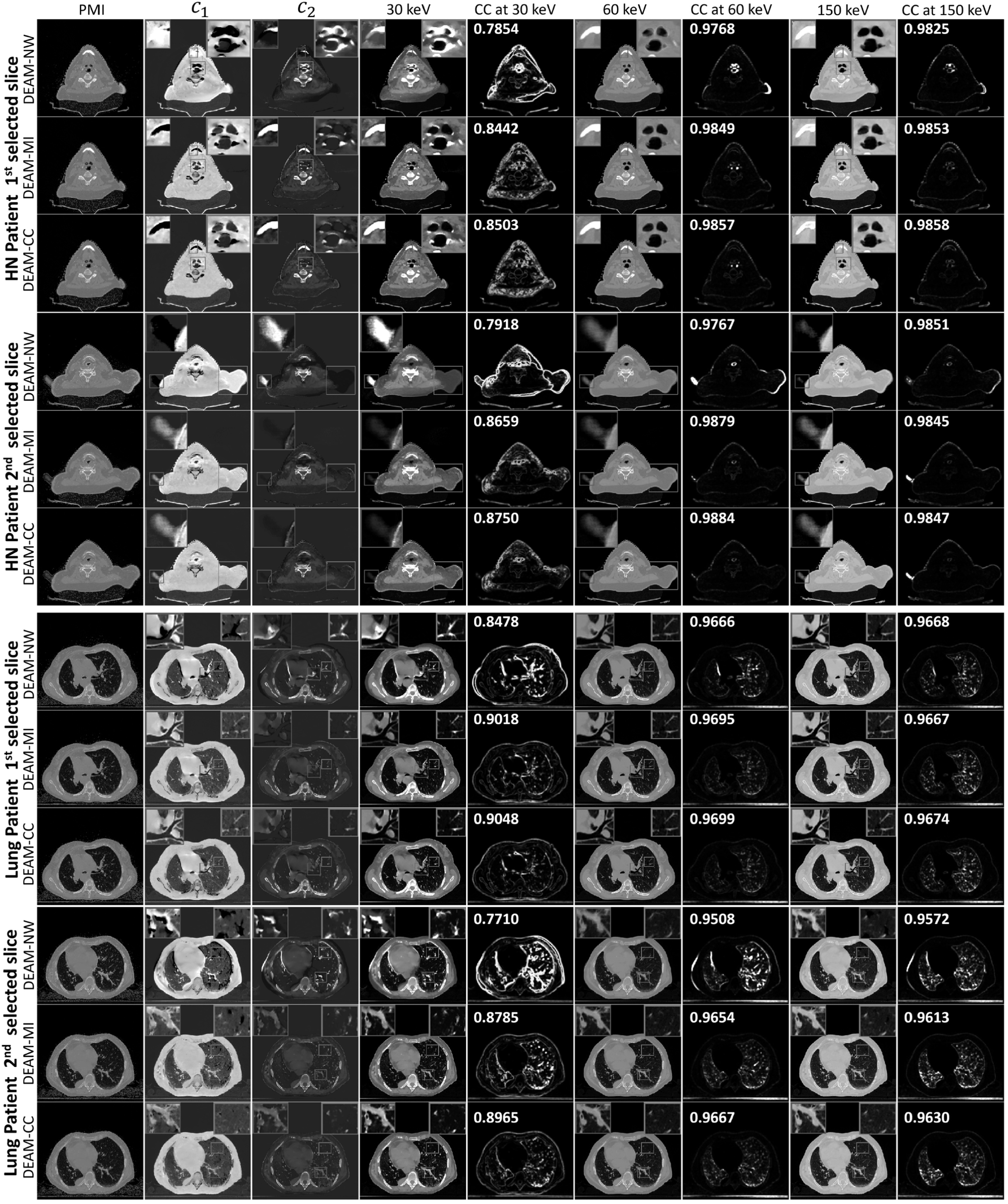
Reconstructed images from non-warped DEAM (DEAM-NW), warped DEAM based on SyN DVFs derived by minimizing the mutual information (DEAM-MI), and cross correlation (DEAM-CC). Two typical slices are selected from a head-neck patient and a lung patient, respectively. Column (1)–(3): inverse PMI overlaying on corresponding 140 kVp image slice, *c*_1_, *c*_2_ (display window [–0.2, 1.2]). Column (4), (6), (8): 30 keV VMI ([0, 0.08]), 60 keV VMI ([0, 0.04]), and 150 keV VMI (display window [0, 0.022]). Column (5), (7), (9): pointwise local cross correlation (PCC) between 140 kVp (fixed) image and 30, 60, 150 keV VMI.

Figure 11 displays reconstructed images by DEAM using different DVFs for two patient datasets. For the head-neck patient, it is observed that the mismatches in the reconstructed images are concentrated in the regions of the pharynx, teeth, and shoulder. These regions of interest are magnified and displayed in the upper left and right corners of the reconstructed images. In the first head-neck slice of the DEAM images, mismatches are observed around the teeth (blue) and pharynx (orange), which exacerbate the artifacts in the DEAM-NW images. In the absence of motion compensation, the pharyngeal wall is reconstructed as a high-density structure in the 30 keV image due to the misalignment of its surface, as shown in the corresponding PMI image. In the second head-neck slice of the DEAM image, the motion artifact on the left shoulder (indicated in blue) has a similar shape to the mismatch identified by the inverse PMI. More mismatches are apparent on the boundary of the right shoulder (indicated in orange), affecting image intensity at the corresponding positions and in the surrounding areas. By incorporating motion compensation, both geometric mismatches (as indicated by PMI image) and the associated image artifacts are significantly reduced.

Due to the lack of the ground truth, we used the 140 kVp image as the structural ground truth, and quantitatively evaluated the image quality through the pointwise cross correlation (PCC) of VMI against the 140 kVp image. PCC images are displayed as 1−PCC, so a brighter region indicates a serverer mismatch. The number on the top right shows the mean value of the corresponding image. Both DEAM-MI and DEAM-CC outperform DEAM-NW with respect to CC images for all keVs, especially for the reconstructions at 30 keV. For the first HN slice, the means of PCC increase from 0.7854 to 0.8442 and 0.8503, and for the second HN slice, from 0.7918 to 0.8659 and 0.8750, respectively.

For the lung dataset, the mismatches are concentrated in the regions of the heart and lung parenchyma blood vessels. These mismatches are manifested as artifactual high-density structures in the 30 keV VMI estimated derived from DEAM-NW (1st, 2nd, 3rd orange and 3rd blue). The blue ROI of the first lung slice shows an overestimation of *c*_1_ and underestimation of *c*_2_ by DEAM-NW, which results in the underestimation of 30 keV attenuation coefficients in the heart. The mismatch and the motion artifact for the right bronchus are shown in the blue rectangles in the second lung slice, which is manifested as anomalously high attenuation coefficients in the bronchial wall. The means of PCC are increased from 0.8478 to 0.9018 (DEAM-MI) and 0.9048 (DEAM-CC) for the first lung slice, and 0.7710 to 0.8785 (DEAM-MI) and 0.8965 (DEAM-CC) for the second lung slice at 30 keV.

In conclusion, the results presented in figure 11 demonstrate that both DEAM-MI and DEAM-CC yield fewer mismatches and motion artifacts than DEAM without registration. Our analysis of the CC images suggests that DEAM with the proposed framework outperforms DEAM without registration, and the DVF derived from minimizing CC outperforms the DVF derived from minimizing MI in this task. This finding is consistent with the research of Brian Avants *et al* (Avants *et al* 2009), who demonstrated that CC is more effective at capturing local patterns and reducing the impact of artifacts and noise than MI.

**Figure 12.**
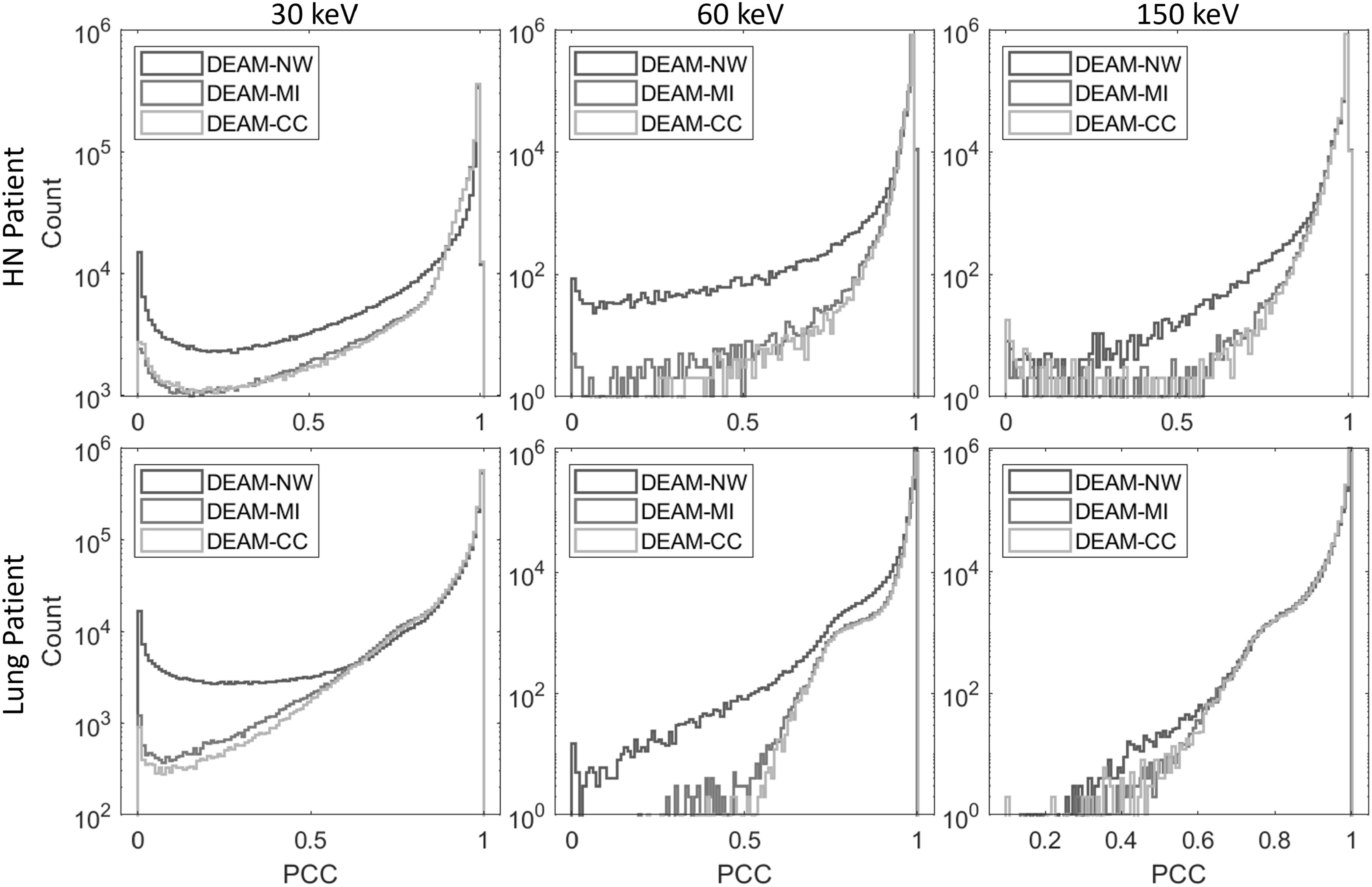
Log-scaled Histograms of PCC. Higher counts of PCC around 1 indicate better performance. First row: head-neck patient, second row: lung patient. From the left column to the right: PCC of 140 kVp image against 30 keV, 60 keV, and 150 keV VMI.

Figure 12 shows the histograms of PCC for clinical imaging reconstructed by DEAM, with and without the use of the DVF. Overall, the PCC values for DEAM-MI and DEAM-CC images exhibit a reduced number of occurrences near zero, indicating a successful reduction of motion artifacts. Additionally, this result also indicates that DEAM-CC outperforms DEAM-MI, as demonstrated by lower PCC counts in the range of 0-0.5.

**Figure 13.**
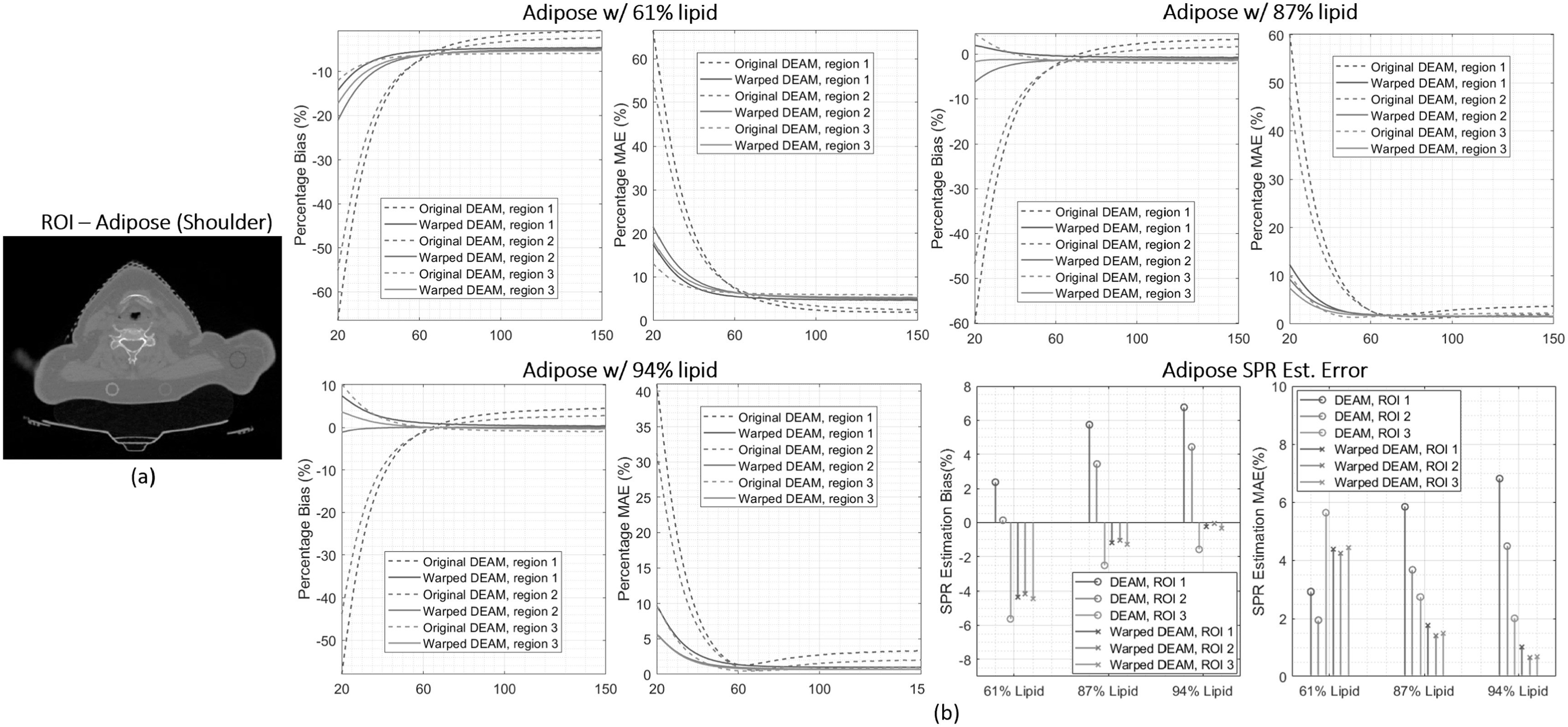
(a) Three adipose regions of interest are indicated by circles. (b) From the first image to the sixth image are the percentage bias and percentage mean absolute error against adipose tissue attenuation with 61%, 87%, and 94% of lipid. The last two figures show the error in estimating SPR.

Figure 13 compares the accuracy with which warped DEAM and uncompensated DEAM algorithms reconstruct LAC and SPRs in three cylindrical adipose ROIs in the head and neck dataset. Since DEAM-CC performed slightly better than DEAM-MI in the previous evaluation, the comparison is limited to DEAM to DEAM-CC. The following quantitative analyzes are based on the assumption that the materials property of the object is close to our anticipation. Since the weight fraction of lipids in adipose tissue is highly variable, we use three different lipid concentrations, 61%, 87%, and 94%, representing the lower limit, mean, and upper limits of lipid mass fraction documented in reference (Woodard and White 1986) to derive three difference adipose-reference LACs.

Three regions of interest in the adipose are indicated in figure 13(a). For the reference adipose with 61%, the magnitude of bias and MAE for warped DEAM is less than the original DEAM result for the 20 to 67 keV energy range above which the uncompensated DEAM outperforms warped DEAM. However, since the bias and MAE in the least affected region (region 3) are larger than the bias and MAE in other regions, it is reasonable to assume that adipose with 61% lipid is unlikely to be the ‘true’ adipose composition for this patient. With the reference adipose with 87% and 94% lipid, the warped DEAM has a lower bias and MAE than the original DEAM at most energies. The percentage MAE with 87% lipid reference adipose is within 2% after 60 keV, and the percentage MAE with 87% lipid reference adipose is within 2% after 47 keV.

**Figure 14.**
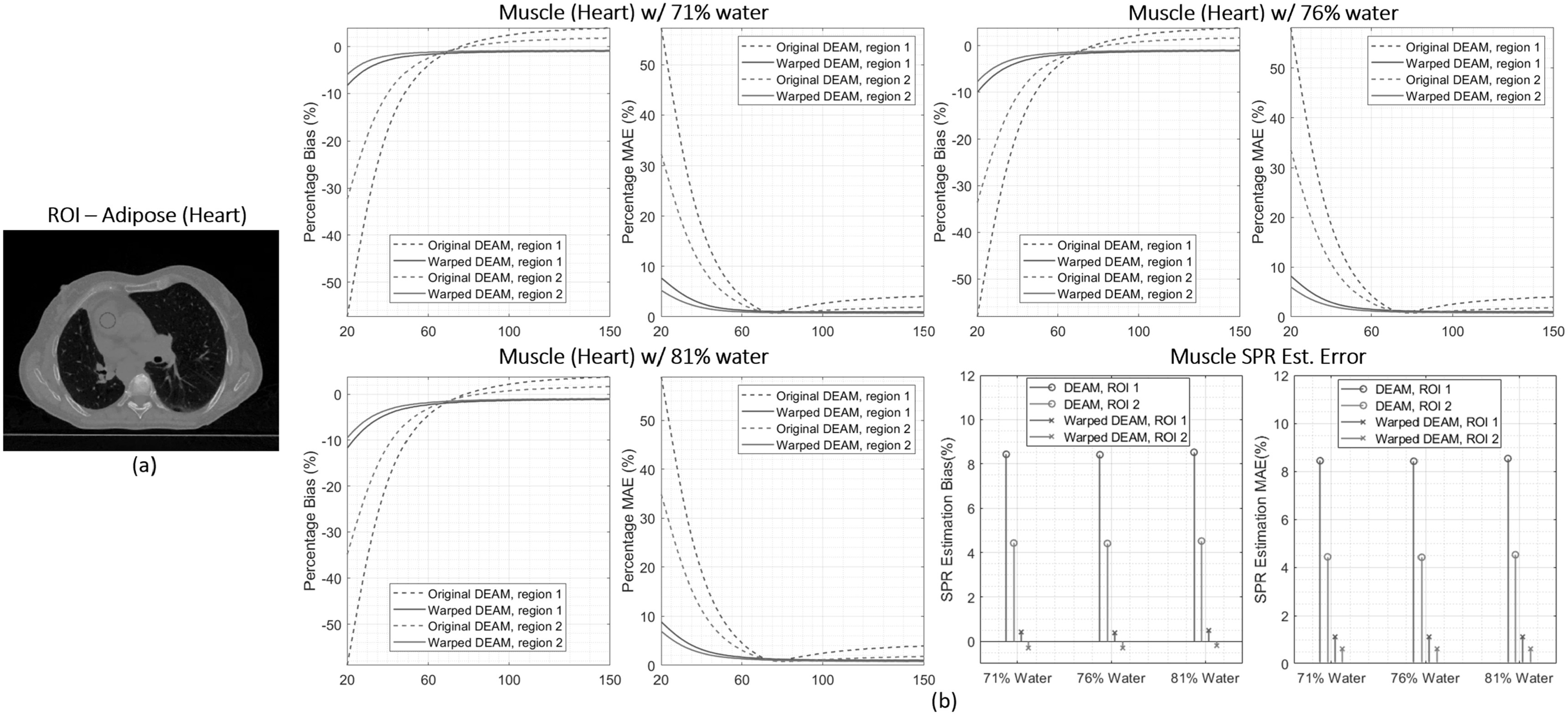
(a) Two adipose regions of interest are indicated by circles. (b) From the first image to the sixth image are the percentage bias and percentage mean absolute error against muscle attenuation with 71%, 76%, and 81% of water. The last two figures show the error in estimating SPR.

Proton SPR estimation error is used as another performance metric. With 87% lipid reference adipose, the SPR bias of original DEAM is 5.7%, 3.4%, and –2.5%, respectively, while the SPR bias of warped DEAM is −1.2%, −1.1%, and −1.2%, respectively. With 94% lipid reference adipose, the magnitude of the SPR bias of warped DEAM could be as low as 0.3%. The same trend could be observed for the plot of MAE. With the proposed scheme, the MAE of DEAM result could be reduced from 5.8%, 3.7%, and 2.8% to 1.7%, 1.4%, and 1.5%. With 94% lipid reference adipose, the MAEs could be as low as 1.0%, 0.6%, and 0.6%. Figure 14 shows a similar analysis of two heart muscle ROIs, where mass fractions of water are varied over the range of documented compositions. Over this range, the variation of muscle LAC is much smaller than for adipose. The magnitude of bias and MAE for images from warped DEAM is less than the corresponding DEAM metrics for all three reference compositions. With the proposed DEAM-CC scheme, the SPR estimation bias is reduced from 8.4% and 4.4% to 0.4% and –0.3%, respectively, while MAE is reduced from 8.4% and 4.4% to 1.1% and 0.6%, respectively.

## 4. Discussion

In this manuscript, we explore the feasibility of utilizing DVFs in DECT SIR to compensate for interscan organ motion and tissue deformation that would otherwise compromise the accuracy of quantitative DECT applications. We fully appreciate that an 8 h reconstruction time is not acceptable for even off-line radiotherapy use cases and continue to investigate acceleration strategies. The DECT SIR process is dominated by the forward and back projection operations, which consume approximately 95% of the elapsed time. In contrast, the motion-correction step is relatively efficient, accounting for less than 1% of the total time as it involves only two trilinear interpolations per iteration. This paper successfully demonstrates that interscan motion corrections can be integrated into the DECT SIR process, enabling accurate imaging of radiological quantities on conventional SECT scanners, without significant loss of either computational efficiency or accuracy.

Nevertheless, the current total reconstruction time still falls far below the clinically acceptable threshold, due to the slow convergence rate of DECT SIR and high computational demands of forward- and back-projection. However, the potential exists to substantially reduce DECT SIR reconstruction time from several hours to on the order of ten minutes through a combination of more efficient update strategies (Degirmenci *et al* 2015, Zhang 2018), deploying additional computational resources (Mitra *et al* 2017), and incorporating deep learning-based acceleration. Recently, a novel model-based deep-learning technique for DECT SIR was proposed (Ge *et al* 2023). The model-based network was able to reconstruct clinical images in less than 6 min, achieving accuracy comparable to existing methods.

Reducing the computational burden associated with accurate radiological quantity mapping remains a challenge, requiring additional engineering efforts to achieve clinically acceptable reconstruction times, especially for online adaptive replanning applications which demand near real-time computational efficiency. However, as currently practiced, proton-treatment planning is an offline non-real-time process (1–2 h), for which a reconstruction time on the order of ten minutes is clinically acceptable. While a two-orders-of-magnitude efficiency gain is a feasible engineering goal, its implementation is beyond the scope of this paper and is left to other ongoing and future investigations by our laboratory.

## 5. Conclusion

We developed a motion-compensated scheme that effectively mitigates motion artifacts in dual-energy sequential scanned reconstructions. This scheme is compatible with any DECT SIR that necessitates the assessment of projection and backprojection. The perturbation analysis described a linear relationship between the error in DVF and the error in the estimated DECT image.

The evaluation of the proposed scheme with a selected DECT SIR, DEAM, indicates a notable decrease in errors related to estimated mono-energetic linear attenuation coefficients. Specifically, our method reduces such errors from a range of 1.45%–21.16% to 0.40%–4.18% in both simulated and clinical cases. In the latter case, we observed significant reductions in motion artifacts in the pharynx, shoulder, and heart regions upon implementation of our scheme.

## Figures and Tables

**Figure 15.**
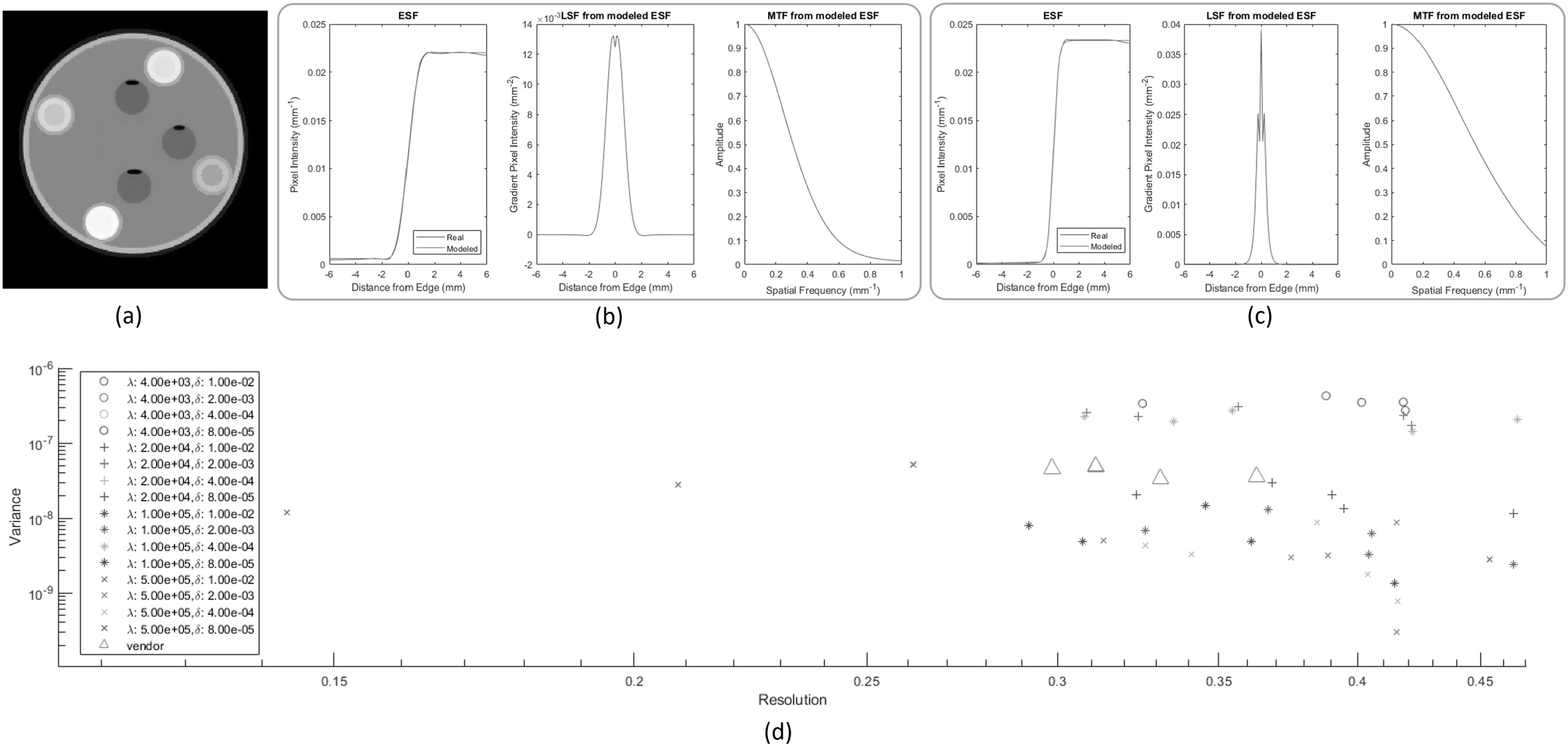
(a) The experimental phantom for the parameter selection study. (b) ESF, LSF, and MTF for vendor reconstruction. (c) ESF, LSF and MTF for DEAM reconstruction with (*λ* = 1*e* ^5^, *δ* = 2*e* ^−3^). (d) scatter plot of variance versus resolution.

## Data Availability

The sinograms from the Philips Big Bore scanner cannot be made publicly available upon publication . because of a confidentiality agreement with Philips Medical Systems (Cleveland) Inc. The data that support the findings of this study are available upon reasonable request from the authors.

## Appendix

### A.1. Derivation of the error analysis

Suppose the objective function of DECT SIR without registration is given by

\begin{eqnarray*}{\ell }(C)=\displaystyle \sum _{j}\displaystyle \sum _{y}{ \mathcal D }\left({d}_{j}(y),{g}_{j}(y:C)\right)+\lambda { \mathcal R }(C).\end{eqnarray*}

Then, the first order necessary condition (FONC) is achieved when

\begin{eqnarray*}\begin{array}{l}-\left(\displaystyle \sum _{j}\displaystyle \sum _{y}\displaystyle \frac{\partial { \mathcal D }}{\partial {g}_{j}(y:C)}\cdot \displaystyle \sum _{E}{I}_{0,j}(y,E)\exp \left(-\displaystyle \sum _{x^{\prime} }h(x^{\prime} ,y)\displaystyle \sum _{i^{\prime} }{\mu }_{i^{\prime} }(E){c}_{i^{\prime} }(x)\right){\mu }_{i}(E)h(x,y)\right)\\ \quad +\lambda \displaystyle \frac{\partial { \mathcal R }(C)}{\partial C}=0\end{array}\end{eqnarray*}

for all *x* and *i*.

Let *C* ^*f*^ be the stationary point of the warped DECT SIR with the truth deformation field *ϕ*_0_ and perfect interpolation mapping ${w}_{{\phi }_{0}}^{0}$. ${\tilde{w}}_{{\phi }_{0}}^{0}$ denotes the perfect adjoint or inverse of ${w}_{{\phi }_{0}}^{0}$. The stationary point is achieved when FONC of the dual-energy transmission problem is satisfied, i.e.

\begin{eqnarray*}\begin{array}{l}-\left(\displaystyle \sum _{y}\displaystyle \frac{\partial { \mathcal D }}{\partial {g}_{H}}{q}_{H,i}(y:{C}^{f})h(x,y)\right)-\left(\displaystyle \sum _{y}\displaystyle \frac{\partial { \mathcal D }}{\partial {g}_{L}}{q}_{L,i}(y:{C}^{f},{w}_{{\phi }_{0}}^{0})\displaystyle \sum _{x}{\tilde{w}}_{{\phi }_{0}}^{0}(x,x^{\prime} )h(x,y)\right)\\ \quad {\left.+\lambda \displaystyle \frac{\partial { \mathcal R }(C)}{\partial C}\right|}_{C={C}^{f}}=0\end{array}\end{eqnarray*}

where *C* ^*f*^ denotes the corresponding stationary point, and

\begin{eqnarray*}{q}_{H,i}(y:{C}^{f})=\displaystyle \sum _{E}{\mu }_{i}(E){I}_{0,H}(y,E)\exp \left(-\displaystyle \sum _{x}h(x,y)\displaystyle \sum _{i^{\prime} }{\mu }_{i^{\prime} }(E){c}_{i^{\prime} }^{f}(x)\right),\end{eqnarray*}

\begin{eqnarray*}{q}_{L,i}(y:{C}^{f},{w}_{{\phi }_{0}}^{0})=\displaystyle \sum _{E}{\mu }_{i}(E){I}_{0,L}(y,E)\exp \left(-\displaystyle \sum _{x}h(x,y)\displaystyle \sum _{x^{\prime} }{w}_{{\phi }_{0}}^{0}(x,x^{\prime} )\displaystyle \sum _{i^{\prime} }{\mu }_{i^{\prime} }(E){c}_{i^{\prime} }^{f}(x^{\prime} )\right).\end{eqnarray*}

Let *w*_*ϕ*_ be an arbitrary interpolation mapping that satisfies ${w}_{\phi }={w}_{{\phi }_{0}}^{0}+{\mathrm{\Delta }}w$, where ∣∣Δ*w*∣∣ → 0 and *ϕ* denotes the estimated deformation field. Assume warped DECT SIR with the above interpolation mapping converges to the stationary point $\hat{C}$. We have

\begin{eqnarray*}\begin{array}{l}-\left(\displaystyle \sum _{y}\displaystyle \frac{\partial { \mathcal D }}{\partial {g}_{H}}{q}_{H,i^{\prime} }(y:\hat{C})h(x,y)\right)-\left(\displaystyle \sum _{y}\displaystyle \frac{\partial { \mathcal D }}{\partial {g}_{L}}{q}_{L,i^{\prime} }(y:\hat{C},{w}_{\phi })\displaystyle \sum _{x}{\tilde{w}}_{\phi }(x,x^{\prime} )h(x,y)\right)\\ \quad {\left.+\lambda \displaystyle \frac{\partial { \mathcal R }(C)}{\partial C}\right|}_{C=\hat{C}}=0\end{array}\end{eqnarray*}

Let $\hat{C}={C}^{f}+{\mathrm{\Delta }}C$, or ${\hat{c}}_{i}={c}_{i}^{f}+{\mathrm{\Delta }}{c}_{i}$ for all *i*. First order Taylor expansion of (A6) at *C* ^*f*^ and ${w}_{{\phi }_{0}}^{0}$ gives

\begin{eqnarray*}\begin{array}{l}-\left(\displaystyle \sum _{y}\displaystyle \frac{\partial { \mathcal D }}{\partial {g}_{H}}{q}_{H,i^{\prime} }(y:{C}^{f})h(x,y)\right)+{\varepsilon }_{H}^{c}({\mathrm{\Delta }}C)\\ \quad -\left(\displaystyle \sum _{y}\displaystyle \frac{\partial { \mathcal D }}{\partial {g}_{L}}{q}_{L,i^{\prime} }(y:{C}^{f},{w}_{{\phi }_{0}}^{0})\displaystyle \sum _{x}{\tilde{w}}_{{\phi }_{0}}^{0}(x,x^{\prime} )h(x,y)\right)\\ {\left.+{\varepsilon }_{L}^{w}({\mathrm{\Delta }}W)+{\varepsilon }_{L}^{c}({\mathrm{\Delta }}C)+\lambda \displaystyle \frac{\partial { \mathcal R }(C)}{\partial C}\right|}_{C={C}^{f}}\\ \quad +\lambda \displaystyle \frac{{\partial }^{2}R(C)}{\partial {C}^{2}}{\mathrm{\Delta }}C+{ \mathcal O }({\mathrm{\Delta }}{W}^{2})+{ \mathcal O }({\mathrm{\Delta }}{C}^{2})=0,\end{array}\end{eqnarray*}

where

\begin{eqnarray*}{\varepsilon }_{H}^{c}({\mathrm{\Delta }}C)=\displaystyle \sum _{y}h(x,y)\displaystyle \sum _{i^{\prime\prime} }{\xi }_{H,{ii}^{\prime} }(y:{C}^{f})\displaystyle \sum _{x^{\prime\prime} }h(x^{\prime\prime} ,y){\mathrm{\Delta }}{c}_{i^{\prime} }(x^{\prime\prime} )\end{eqnarray*}

\begin{eqnarray*}\begin{array}{rcl}{\varepsilon }_{L}^{c}({\mathrm{\Delta }}C) &amp; = &amp; \displaystyle \sum _{x}{\tilde{w}}_{{\phi }_{0}}(x,x^{\prime} )\displaystyle \sum _{y}h(x,y)\displaystyle \sum _{i^{\prime\prime} }{\xi }_{L,{ii}^{\prime} }(y:{C}^{f},{w}_{{\phi }_{0}}^{0})\\ &amp; &amp; \cdot \displaystyle \sum _{x^{\prime\prime} }h(x^{\prime\prime} ,y)\displaystyle \sum _{x\prime\prime\prime }{w}_{{\phi }_{0}}^{0}(x^{\prime\prime} ,x\prime\prime\prime )\displaystyle \sum _{i^{\prime} }{\mathrm{\Delta }}{c}_{i^{\prime} }(x\prime\prime\prime )\end{array}\end{eqnarray*}

\begin{eqnarray*}\begin{array}{rcl}{\varepsilon }_{L}^{w}({\mathrm{\Delta }}W) &amp; = &amp; \displaystyle \sum _{x}{\tilde{w}}_{{\phi }_{0}}^{0}(x,x^{\prime} )\displaystyle \sum _{y}h(x,y)\displaystyle \sum _{i^{\prime\prime} }{\xi }_{L,{ii}^{\prime} }(y:{C}^{f},{w}_{{\phi }_{0}}^{0})\\ &amp; &amp; \cdot \displaystyle \sum _{x^{\prime\prime} }h(x^{\prime\prime} ,y)\displaystyle \sum _{x\prime\prime\prime }{\mathrm{\Delta }}w(x^{\prime\prime} ,x\prime\prime\prime ){\hat{c}}_{i^{\prime} }(x\prime\prime\prime )\\ &amp; &amp; -\displaystyle \sum _{x}\displaystyle \sum _{x^{\prime\prime} }\displaystyle \sum _{x\prime\prime\prime }{\mathrm{\Delta }}w(x^{\prime\prime} ,x\prime\prime\prime )\displaystyle \frac{\partial {\tilde{w}}_{{\phi }_{0}}^{0}(x,x^{\prime} )}{\partial {w}_{{\phi }_{0}}^{0}(x^{\prime\prime} ,x\prime\prime\prime )}\displaystyle \sum _{y}h(x,y)\displaystyle \frac{\partial { \mathcal D }}{\partial {g}_{L}}{q}_{L,i}(y:{C}^{f},{w}_{{\phi }_{0}}^{0})\end{array}\end{eqnarray*}

\begin{eqnarray*}{\xi }_{H,{ii}^{\prime} }(y:{C}^{f})=\displaystyle \frac{{\partial }^{2}{ \mathcal D }}{\partial {g}_{H}^{2}}\displaystyle \sum _{E}{\mu }_{i}(E){\mu }_{i^{\prime} }(E){I}_{0,H}(y,E)\exp \left(-\displaystyle \sum _{x}h(x,y)\displaystyle \sum _{i^{\prime\prime} }{\mu }_{i^{\prime\prime} }(E){c}_{i^{\prime\prime} }^{f}(x)\right),\end{eqnarray*}

\begin{eqnarray*}\begin{array}{rcl}{\xi }_{L,{ii}^{\prime} }(y:{C}^{f},{w}_{{\phi }_{0}}^{0}) &amp; = &amp; \displaystyle \frac{{\partial }^{2}{ \mathcal D }}{\partial {g}_{L}^{2}}\displaystyle \sum _{E}{\mu }_{i}(E){\mu }_{i^{\prime} }(E){I}_{0,L}(y,E)\\ &amp; &amp; \exp \cdot \left(-\displaystyle \sum _{x}h(x,y)\displaystyle \sum _{x^{\prime} }{w}_{{\phi }_{0}}^{0}(x,x^{\prime} )\displaystyle \sum _{i^{\prime\prime} }{\mu }_{i^{\prime\prime} }(E){c}_{i^{\prime\prime} }^{f}(x^{\prime} )\right).\end{array}\end{eqnarray*}

Combine (A6) and (A7),

\begin{eqnarray*}{\varepsilon }_{L}^{w}({\mathrm{\Delta }}W)+{\varepsilon }_{L}^{c}({\mathrm{\Delta }}C)+{\varepsilon }_{H}^{c}({\mathrm{\Delta }}C)+\lambda \displaystyle \frac{{\partial }^{2}{ \mathcal R }(C)}{\partial {C}^{2}}{\mathrm{\Delta }}C+{ \mathcal O }({\mathrm{\Delta }}{W}^{2})+{ \mathcal O }({\mathrm{\Delta }}{C}^{2})=0\end{eqnarray*}

More succinctly,

\begin{eqnarray*}\left(\tilde{W}{H}^{\dagger }{{\mathrm{\Lambda }}}_{L}{HW}+{H}^{\dagger }{{\mathrm{\Lambda }}}_{H}H+\lambda {\mathrm{\Psi }}\right){\mathrm{\Delta }}C\approx {\mathrm{\Omega }}({\mathrm{\Delta }}W){HQ}-\tilde{W}{H}^{\dagger }{{\mathrm{\Lambda }}}_{L}H{\mathrm{\Delta }}{WC}\end{eqnarray*}

where *C* = [*c*_1_; *c*_2_] is a 2*N*_*x*_ × 1 vector, ${{\mathrm{\Lambda }}}_{i}\in {{\mathbb{R}}}^{2{N}_{y}\times 2{N}_{y}}$ consists of four diagonal matrices such that ${\left[{{\mathrm{\Lambda }}}_{L}\right]}_{i,i^{\prime} }(y,y)={\xi }_{L,{ii}^{\prime} }(y:{C}^{f},{w}_{{\phi }_{0}}^{0})$ and ${\left[{{\mathrm{\Lambda }}}_{H}\right]}_{i,i^{\prime} }(y,y)={\xi }_{H,{ii}^{\prime} }(y:{C}^{f})$, ${\mathrm{\Psi }}=\tfrac{{\partial }^{2}{ \mathcal R }(C)}{\partial {C}^{2}}$, ${\mathrm{\Omega }}({\mathrm{\Delta }}W)=\left[\begin{array}{cc}1 &amp; 0\\ 0 &amp; 1\end{array}\right]\otimes \tilde{{\mathrm{\Omega }}}({\mathrm{\Delta }}W)$, where ⨂ denotes the Kronecker product, and $[\tilde{{\mathrm{\Omega }}}({\mathrm{\Delta }}W)](x,x^{\prime} )={\sum }_{x^{\prime\prime} }{\sum }_{x\prime\prime\prime }{\mathrm{\Delta }}w(x^{\prime\prime} ,x\prime\prime\prime )\tfrac{\partial {\tilde{w}}_{{\phi }_{0}}^{0}(x,x^{\prime} )}{\partial {w}_{{\phi }_{0}}^{0}(x^{\prime\prime} ,x\prime\prime\prime )}$.*Q* is a 2*N*_*y*_ × 1 vector whose entry is $Q(y)={q}_{L,i}(y:{C}^{f},{w}_{{\phi }_{0}}^{0})$.

### A.2. Gradient evaluation of local penalty

In this appendix, the derivative of the local potential function $\psi \left({c}_{i}(x)-{c}_{i}(x^{\prime} )\right)$ for image update is derived from its convex property. A function *f*(*t*) is convex if and only if

\begin{eqnarray*}f(\alpha {t}_{1}+(1-\alpha ){t}_{2})\leqslant \alpha f({t}_{1})+(1-\alpha )f({t}_{2}),\quad \forall \alpha \in [0,1].\end{eqnarray*}

Therefore,

\begin{eqnarray*}\begin{array}{l}\psi \left({c}_{i}(x)-{c}_{i}(x^{\prime} )\right)=\psi \left(\displaystyle \frac{1}{2}\left(2\left({c}_{i}(x)-{\hat{c}}_{i}(x)\right)+\left({\hat{c}}_{i}(x)-{\hat{c}}_{i}(x^{\prime} )\right)\right)\right.\\ \quad \left.+\displaystyle \frac{1}{2}\left(-2\left({c}_{i}(x^{\prime} )-{\hat{c}}_{i}(x^{\prime} )\right)+\left({\hat{c}}_{i}(x)-{\hat{c}}_{i}(x^{\prime} )\right)\right)\right)\end{array}\end{eqnarray*}

\begin{eqnarray*}\begin{array}{l}\leqslant \displaystyle \frac{1}{2}\psi \left(2{c}_{i}(x)-2{\hat{c}}_{i}(x)+\left({\hat{c}}_{i}(x)-{\hat{c}}_{i}(x^{\prime} )\right)\right)\\ \quad +\displaystyle \frac{1}{2}\psi \left(2{c}_{i}(x^{\prime} )-2{\hat{c}}_{i}(x^{\prime} )+\left({\hat{c}}_{i}(x)-{\hat{c}}_{i}(x^{\prime} )\right)\right)\end{array}\end{eqnarray*}

\begin{eqnarray*}=\displaystyle \frac{1}{2}\psi \left(2{c}_{i}(x)-{\hat{c}}_{i}(x)-{\hat{c}}_{i}(x^{\prime} )\right)+\displaystyle \frac{1}{2}\psi \left(2{c}_{i}(x^{\prime} )-{\hat{c}}_{i}(x^{\prime} )-{\hat{c}}_{i}(x)\right),\end{eqnarray*}

and the derivative of the surrogate penalty is

\begin{eqnarray*}\displaystyle \frac{\partial }{\partial {c}_{i}(x)}\hat{R}\left({c}_{i}(x)-{c}_{i}(x^{\prime} )\right)=\displaystyle \sum _{x^{\prime} \in N(x)}w(x,x^{\prime} ){\left.\displaystyle \frac{\partial \psi (t)}{\partial t}\right|}_{t=2{c}_{i}(x)-{\hat{c}}_{i}(x)-{\hat{c}}_{i}(x^{\prime} )}.\end{eqnarray*}

### A.3. Parameter selection of regularization term

The variance and resolution are considered as the image metrics to select the optimal penalty parameter. For traditional computational imaging, there is a trade-off between the strength of noise reduction and the image resolution.

We use a cylinder phantom with cylinder inserts to evaluate the influence of the penalty parameter values on the selected two image metrics. The transmission data is acquired from a Philips Big Bore scanner at the same dose level as the patient data. The resolution evaluation process follows the steps in (Evans *et al* 2011). Pixels that share the same distance to the center are accumulated in the same bin to produce the edge spread function (ESF). Then, a nonlinear data-fitting solver is used to model the discretized ESF as a continuous function

\begin{eqnarray*}{ESF}(r)=e+f\cdot \mathrm{sign}(r)\cdot (a\cdot (1-{e}^{-b| r| }))+c\cdot \mathrm{erf}(d\cdot | r| ),\end{eqnarray*}

where *a*, *b*, *c*, *d*, *e*, *f* are parameters to be estimated.

The line spread function is given by

\begin{eqnarray*}{LSF}(r)=\displaystyle \frac{d}{{dr}}{ESF}(r)=f\cdot ({ab}\cdot ({e}^{-b| r| }))+{cd}\cdot \displaystyle \frac{2}{\sqrt{\pi }}{e}^{-{d}^{2}{r}^{2}},\end{eqnarray*}

and the modulation transfer function (MTF) is therefore

\begin{eqnarray*}{MTF}(\tilde{\omega })=| {FT}({LSF})| =f\cdot \left(\displaystyle \frac{2{{ab}}^{2}}{{b}^{2}+4\pi \tilde{\omega }}+2c\cdot {e}^{-\tfrac{{\left(\pi \tilde{\omega }\right)}^{2}}{{d}^{2}}}\right).\end{eqnarray*}

The resolution metric is calculated by the integral from the frequency 0 to 0.5, i.e.

\begin{eqnarray*}{\int }_{0}^{0.5}{MTF}(\tilde{\omega })d\tilde{\omega },\end{eqnarray*}

and the noise level metric is denoted by the variance of the selected uniform region.

In figure 15(a), five red circles indicate five selected edges for ESF evaluation, and the corresponding variance is computed on the region surrounded by each circle. Figure 15(b) and (c) show two examples of ESF, LSF, and MTF for the Vendor and DEAM images. Figure 15(d) shows the scatter plot of variance versus resolution of the DEAM result with different sets of penalty parameters compared with the vendor reconstruction. We selected (*λ* = 1*e* ^5^, *δ* = 2*e* ^−3^) as the parameter set for DEAM because DEAM with this set of penalty parameters generates images with higher resolution and less noise compared to the vendor reconstruction.
